# Molecular cloning and expression analysis of three *ThERF*s involved in the response to waterlogging stress of *Taxodium* ‘Zhongshanshan406’, and subcellular localization of the gene products

**DOI:** 10.7717/peerj.4434

**Published:** 2018-03-12

**Authors:** Wencai Fan, Ying Yang, Zhiquan Wang, Yunlong Yin, Chaoguang Yu, Qin Shi, Jinbo Guo, Lei Xuan, Jianfeng Hua

**Affiliations:** Institute of Botany, Jiangsu Province and Chinese Academy of Sciences, Nanjing, Jiangsu, China

**Keywords:** *ThERF* genes, *Taxodium* hybrid ‘Zhongshanshan 406’, Cloning, Subcellular localization, Expression analysis, Ethylene concentration

## Abstract

As a subfamily of the APETALA 2/ethylene response element binding protein (AP2/EREBP) transcription factor superfamily, the ethylene response factor (ERF) is widely involved in the regulation of growth and response to various abiotic stresses in plants, and has been shown to be the main transcription factor regulating transcription of the genes related to hypoxia and waterlogging stress. In this study, three *ThERF* genes, with significant differences in expression profile in response to flooding stress, were identified from the transcriptomics data acquired from *Taxodium* hybrid ‘Zhongshanshan 406’ (*T. mucronatum* Tenore × *T. distichum* (L.) Rich) under waterlogging stress: *ThERF15, ThERF39 and ThRAP2.3* (GenBank ID: KY463467, KY463468 and KY463470, respectively).The full-length cDNA of each of the three *ERF*s was obtained using the RACE (rapid amplification cDNA ends) method, and all three were intron-free. Multiple protein sequence alignments indicated that ThERF15, ThERF39 and ThRAP2.3 proteins all had only one AP2-ERF domain and belonged to the ERF subfamily. A transient gene expression assay demonstrated that ThERF15, ThERF39 and ThRAP2.3 were all localized to the nucleus. Real-time quantitative PCR (qPCR) revealed that the expression of *ThERF15, ThERF39 and ThRAP2.3* exhibited significant differences, compared with the control, in response to two levels of flooding treatment (half-flooding or total-submergence) of ‘Zhongshanshan 406’. Quantification of ethylene concentration revealed that ethylene was more relevant to the level of expression than the period of flooding treatment. Based on the experimental results above, *ThERF15, ThERF39* and *ThRAP2.3* were identified as being related to the regulation of downstream flooding- responsive gene expression in ‘Zhongshanshan 406’. *ThRAP2.3* is most likely to be a key downstream-response ERF gene to respond to the output of the ethylene signal generated by flooding stress.

## Introduction

In China, there is great potential for the development of afforestation in water-logged areas, due to the abundant resources of shallows and wetlands. Further research on tree species with high waterlogging tolerance would be beneficial to improve the utilization of land resources in coastal areas and wetlands in China. Afforestation is also an important measure to solve the problems of flood disaster, biodiversity decline and wetland restoration along the coastal and riverside areas of China (Cao et al., 2010).

To date, compared with herbaceous plants, fewer xylophyta have been reported to exhibit long-term flooding tolerance (Wang et al., 2016; Yu et al., 2012). The genus *Taxodium* is well known for its waterlogging tolerance and landscape values (Conner, Mihalia & Wolfe, 2002; Souther & Shaffer, 2000). Interspecific hybridization in *Taxodium* Rich can combine the best characteristics of superior parents (Zhou et al., 2010). *Taxodium* hybrid ‘Zhongshanshan406’, which is an elite clone selected from the hybrid *Taxodium mucronatum* ♀ ×*Taxodium distichum* ♂, developed by Institute of Botany, Jiangsu Province and Chinese Academy of Sciences, showed great improvement in flooding tolerance, and has been widely planted in China (including Dianchi Lake in Yunnan Province, Chaohu City in Anhui Province, Three-Gorge reservoir in Chongqing Province) as a wetland species (Han & Shan, 2012; Ma et al., 2011). Related physiological and transcriptomics studies have demonstrated that ‘Zhongshanshan406’ is an ideal model plant with which to research the waterlogging characteristics of woody plants (Hua et al., 2017; Qi et al., 2014).

To cope with abiotic stresses, including waterlogging stress, transcription factors (TFs) play a central role by regulating expression of downstream stress-responsive genes via sequence-specific binding to *cis*-acting elements in the promoters of target genes (Hussain, Kayani & Amjad, 2011; Mizoi, Shinozaki & Yamaguchi-Shinozaki, 2012). As a large superfamily of plant-specific transcription factors, the APETALA2/ethylene-responsive binding factor (AP2/EREBP) family is involved in a myriad of regulatory processes, such as growth and development, metabolic regulation, and response to a variety of biological and abiotic stresses (Upadhyay et al., 2013). The AP2/EREBP family includes four major subfamilies: the AP2 (APETALA2), DREB (dehydration-responsive element-binding protein), RAV (related to ABI3/VP1) and ERF (ethylene-response factor) subfamilies (Zhuang et al., 2009). Of these, many ERF subfamily members have been isolated and characterized with respect to their major effects, related to responses to plant hypoxia and flooding stress. These ERFs function by regulating gene expression of downstream stress-tolerance genes via the *cis*-acting ethylene-responsive element (ERE), known as the GCC box (AGCCGCC; Xu et al., 2007). All the ERF subfamily members have a conserved 58–59 amino acid AP2/ERF domain, which makes them capable of performing the above adjustment functions (Fujimoto et al., 2000; Mizoi, Shinozaki & Yamaguchi-Shinozaki, 2012).

Ethylene is a key upstream regulatory component of the ethylene signal transduction pathway, its effects being mediated by signal transduction components, including ERF transcription factor (TF) families (Yin et al., 2012). Ethylene plays a critical role in myriad developmental programs and fitness responses to pathogens and abiotic stress factors (Alonso & Stepanova, 2004; Guo & Ecker, 2004). Many studies have shown that a series of ethylene receptors (ETR1, ETR2, ERS and EIN4), located in the endoplasmic reticulum membrane, can interact with the ethylene molecule to exert inhibitory effects on the kinase activity of CTR1 (constitutive triple response 1), to release the key positive regulator EIN2(ethylene insensitive 2) of the ethylene signaling pathway, and then send the signals toEIN3/EIL1transcriptional regulatory factors and downstream *ERF*-responsive genes, thus completing the output of the ethylene signal (Bisson & Groth, 2011; Ju et al., 2012; Li, Ma & Guo, 2013).

Despite numerous reports on the ERF subfamilies in herbaceous model plants (Joo et al., 2013; Licausi et al., 2010a; Licausi et al., 2010b), there has been little progress on the isolation and characterization of the *ERF* genes operating in response to flooding and hypoxia stresses in woody plants, with no reports from *Taxodium* hybrid ‘Zhongshanshan’. Our objectives of this work were to explore the molecular mechanisms of the *ThERF*s to tolerate waterlogging stress and it’s vital for us because it’s a research gap in ‘Zhongshanshan’. In this study, we identified and characterized three genes encoding ERF proteins (ThERF15, ThERF39 and ThRAP2.3) from ‘Zhongshanshan 406’, and explored their expression profiles, in comparison with the ethylene-accumulation mechanism in waterlogging stress, hoping to enrich the genetic resources of the ERF TFs in ‘Zhongshanshan 406’, and to make a preliminary exploration of the molecular mechanisms involved in the accumulation of ethylene at the protein level.

## Materials and Methods

### Plant materials

One-year-old cuttings of ‘Zhongshanshan 406’ plants were obtained from Institute of Botany, Jiangsu Province and Chinese Academy of Science, Nanjing, China. Prior to the experiment, about 100 healthy ‘Zhongshanshan 406’ plants were carefully transplanted into plastic pots containing 3:1:1 (v/v/v) clay, vermiculite and perlite in July 2016. Each pot was 20 cm in diameter and 25 cm in height. All plants were irrigated fully every two days. A pot tray was placed under each pot to avoid water loss and soil erosion. The plants were allowed to acclimate to the local conditions for 60 days before the treatments were imposed.

In early September, 90 plants were selected on the basis of uniformity of size and development. One of three treatments was applied to each batch of thirty plants: non-flooding (control, CK), half-flooding (HF) and total-submergence (TS).The plants with treatments HF and TS were put into outdoor concrete tanks (2.8 m × 1.7 m each) filled with tap water to different depths. CK plants were placed near the tanks and watered normally. HF plants were flooded to a water level 5 cm above the soil surface. TS plants were completely submerged. On the first, third, fifth, seventh, and ninth days of the treatments, leaf tissue and root tissue (for treatments CK, HF and TS) were sampled for analysis at each of these five time points, the tissue being immediately snap-frozen in liquid nitrogen and stored at −80 °C until extraction of RNA and DNA. Three independent biological replicates were sampled at each time point for each treatment.

### Determination of ethylene content

Tissues and time points for sampling were the same as above. For each time point, the leaf tissue and the root tissue were sampled from the three treatments and five biological replicates were prepared for each sample. The tissue in each replicate weighed 0.2 g, and ethylene was extracted from the tissue and assayed using the Plant ETH ELISA Kit (HCB, Vancouver, Canada). The concentration of ethylene in each replicate was determined by comparing the absorbance of the samples to a standard curve.

### RNA extraction and cDNA synthesis

The frozen plant tissues for RNA extraction were ground into a fine powder using a mortar and pestle. Total RNA was extarcted from the leaves or roots from the CK, HF and TS treatments at the five sampling time points, using the RNeasy^®^ Plant Mini kit (Qiagen, Dusseldorf, Germany), and was reverse transcribed with iScript™ cDNA Synthesis Kit (BIO-RAD, Hercules, CA, USA), using 1 mg RNA as the template.

### Molecular cloning

On the basis of the sequence fragments obtained from the transcriptomics data, which was obtained from ‘Zhongshanshan 406’ plants under waterlogging stress, nested primers were designed using the Oligo software (Version 6.0) to amplify the full-length sequences with the 3′-Full RACE Core Set Kit (TaKaRa, Otsu, Japan) and SMATer RACE 5′/3′ Kit (Clontech, Palo Alto, CA, USA), according to the manufacturer’s instructions. The amplified fragments were separated on 1% agarose gels and purified by QIAquick Gel Extraction Kit (QIAGEN, Dusseldorf, Germany), linked into pMD19-T vectors (TaKaRa, Otsu, Japan) and finally transformed into *Escherichia coli* strain TOPIO. The recombinant plasmids were checked by PCR, and the positive colonies were sequenced. The overlapping sequences were assembled by BioEdit software (Version 2.6) and the full-length cDNA sequences of the three *ERFs* were obtained. The predicted open reading frames (ORFs) were subsequently amplified by PCR, and were verified by sequencing. Genomic DNA was extracted using a Plant Genomic DNA Extraction Kit (BioTeke, Beijing, China). Genomic DNA sequences of the above genes were amplified with the RNase-treated DNA, and were verified by sequencing. The sequences of the primers are listed in Table 1.

**Table 1:** Primer sequences of the genes used in the research.

Prime-ID	Forward PCR Primer(5′–3′)	Reverse PCR Primer(5′–3′)
ThERF15_3OUTER	AAAGAAAGCATTACAGAGGCGTG	AAATCACTAGTGGAACGACGGTA
ThERF15_3INNER	CAGTACCAGTACCAAATACCGAG GGTTGAGAACGAG	CCTATAGTGAAATCACTAGTGGAGGATCCGCG
ThERF15_5OUTER	CTAATACGACTCACTATAGGGCAAGCAGTGGT ATCAACGCAGAGT	TTTTTCCTCCATTTGCTCGTTCTCAA
ThERF15_5INNER	CTAATACGACTCACTATAGGGC	TCTGATTTCCGCAGCAAACTTTC
ThERF15_ORF	ATGGCGCAATTGAAGAGTA	GTTAAGCTGTAAGTCACATGAG
ThERF15_qRT-PCR	GGGATTTGAGGGTAAAAAAG	GATGCCGATTCTCTGATTTC
ThERF39_3OUTER	AAGATGGGCTGCTGAGATAAGAG	AAATCACTAGTGGAACGACGGTA
ThERF39_3INNER	GCAAATGCCAAGACGAAGCCCAATACAATAGA	CCTATAGTGAAATCACTAGTGGAGGATCCGCG
ThERF39_5OUTER	CTAATACGACTCACTATAGGGCAAGCA GTGGTATCAACGCAGAGT	GCTTTCTATTGTATTGGGCTTCGTCTT
ThERF39_5INNER	CTAATACGACTCACTATAGGGC	ACCAGACAAAGTTATTGGGAGCAGAGG
ThERF39_ORF	ATGAAGTATGAGTACTCACCAGA	TACATTTATCCAGCTCAGAGT
ThERF39_qRT-PCR	GGAGTTAGAGTATGGTTGGG	AAAGTTATTGGGAGCAGAGG
ThRAP2.3_3OUTER	GGACAAGGTGAATGTCTCTGTTC	AAATCACTAGTGGAACGACGGTA
ThRAP2.3_3INNER	TGCCACACAACAGTGCTTCTCTCTGGGTTT	CCTATAGTGAAATCACTAGTGGAGGATCCGCG
ThRAP2.3_5OUTER	CTAATACGACTCACTATAGGGCAA GCAGTGGTATCAACGCAGAGT	TCTTTTCTTTTCATAATTTGCAGCAGG
ThRAP2.3_5INNER	CTAATACGACTCACTATAGGGC	TGCTTCCTCCACACATTTTCTCTTTG
ThRAP2.3_ORF	ATGACGGTAAAAAGCGGAG	AAAGATTGCAGTCCACAGA
ThRAP2.3_qRT-PCR	CAACTCAGTGGAAGATGCTG	CACCTTGTCCGTAGATTTGT
APRT_qRT-PCR	TCCACAGGTTCTTGAATCGCT	TGACTTGAGCCTCATTCGCTC

### Bioinformatics and statistical analyses

The online BLAST software (https://blast.ncbi.nlm.nih.gov/Blast.cgi) was used to analyze the DNA and protein sequences of the three ERFs. ORFs were predicted by DNAMAN software (Version 8.0). The physicochemical properties and amino acid composition of the proteins were predicted and calculated using Expasy Protparam (http://web.expasy.org/protparam/). Analyses of the signal peptide cleavage site, protein domain search and transmembrane structures of the genes were carried out with SignalP online tools (http://www.cbs.dtu.dk/services/SignalP/), PROSITE (http://prosite.expasy.org/) and TMHMM (http://www.cbs.dtu.dk/services/TMHMM/), respectively. GOR IV secondary structure prediction method (https://npsa-prabi.ibcp.fr/cgi-bin/npsa_automat.pl?page=npsa_gor4.html) was used to predict secondary structures of the deduced amino acid sequences. Alignment of the deduced protein sequences of *ThERF15, ThERF39 and ThRAP2.3* with other plant ERF sequences was performed using the ClustalX software (http://www.clustal.org/clustal2/). For the analysis of evolutionary relationships, phylogenetic trees were constructed, employing the Neighbor-Joining (NJ) method with 1,000 bootstrap replicates, using the MEGA 7 software (http://www.megasoftware.net/) (Kumar, Stecher & Tamura, 2016). Data of ethylene concentration approximated to normality, and was analyzed with one-way analysis of variance (ANOVA) followed by multiple comparisons using Duncan’s multiple range test at *P* = 0.05, using SPSS 19.0 software (SPSS Inc., Chicago, IL, USA).

### Subcellular localization

Transient expression vectors of the three *ERFs* were constructed using the TOPO and Gateway technologies (Invitrogen, Carlsbad, CA, USA). The coding DNA sequence (CDS) regions were inserted into the entry vectors, pCR™ 8/GW/TOPO™ (Invitrogen, Carlsbad, CA, USA). Then, the inserts from the entry vectors were transferred to the destination vectors (p2GWF7) and confirmed by sequencing. The plant expression vectors (35S::*ThERF15*-GFP, 35S::*ThERF39*-GFP and 35S::*ThRAP2.3*-GFP) which containing the green-fluorescence protein (GFP) were obtained. Protoplast isolation and polyethylene glycol-mediated transfection were performed by the method of Tan et al. (2013). Nucleus were stained with 4′,6-diamidino-2-phenylindole (DAPI, 1 ug ml^−1^, Sigma). The fluorescent signals were observed by a BX51 173 fluorescence microscope (Olympus).

### Real-time qPCR analyses

To quantify the expression of *ThERF15, ThERF39 and ThRAP2.3*, qPCR was performed on an Analytik Jena qTOWER2.2 PCR System (Biometra, Gottingen, Germany). The primers for the three *ERFs* (Table 1) for qPCR were designed, based on the sequence of their cDNA. Adenine phosphor ribosyl transferase (*APRT*, GenBank accession No. KX431853) was used as a reference gene, which was amplified using the primers APRT-F and APRT-R (Table 1). Each sample was carried out in triplicate. The results are displayed in the form of relative expression values 2^−ΔΔCt^, where ΔCt represents the Ct value of the gene minus that of the reference gene (Bustin et al., 2009; Livak & Schmittgen, 2001).

## Results

### Ethylene concentration

**Figure 1:**
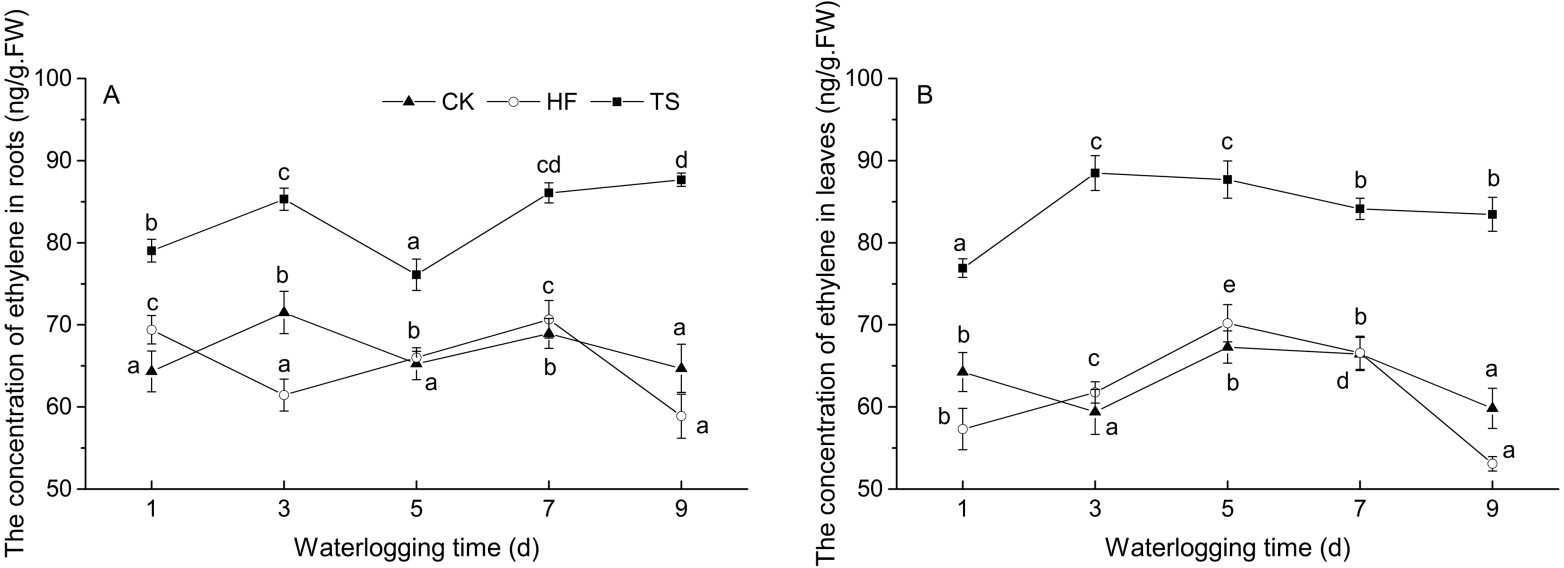
The change of ethylene concentrations under CK, HF and TS treatments. Data points are the mean values and error bars represent the standard errors, which are from five biological replicates. (A) in roots; (B) in leaves. The difference of letters at the top of the line shows that there is a significant difference in ethylene content at different waterlogging times under the same degree of flooding treatment (*P* < 0.05).

Compared with the HF and TS treatment groups, the concentration of ethylene in the CK group fluctuated within a relatively narrow range during the whole treatment process. In both roots and leaves, ethylene production was higher in the TS treatments, compared to the CK, whereas ethylene production in HF and CK were generally not significantly different in either roots or leaves. In both leaves and roots, the lowest ethylene concentrations occurred in the HF treatment on day 9, whereas such a decline was not observed in TS (Fig. 1).

### Cloning and characterization of the *ERF* genes

Three ethylene-response factor genes, *ThERF15, ThERF39 and ThRAP2.3*, which belong to the ERF transcription factor family, were cloned from the cDNA of ‘Zhongshanshan 406’, using the RACE technique. Comparisons of the genomic DNA (gDNA) and copy DNA (cDNA) sequences indicated that each of the three *ERFs* was intron-free. Nucleotide sequences revealed that the full-length cDNA of *ThERF15* was 1,036 bp, containing a 23 bp 5′-untranslated region (UTR), a 194 bp 3′UTR and a 819 bp open reading frame (ORF), which encoded a deduced protein of 272 amino acids. The full-length cDNA of *ThERF39* was 1,723 bp, with a predicted ORF of 1,137 bp, flanked by a 95 bp 5′UTR and a 491 bp 3′UTR, and encoding a deduced protein of 378 amino acids. *ThRAP2.3* comprised of a predicted ORF of 1,107 bp, flanked by a 196 bp 5′UTR and a 107 bp 3′UTR (full-length cDNA was 1,410 bp), the ORF encoding a deduced protein of 368 amino acids (Table 2).

**Table 2:** Characterization of the genes *ThERF15, ThERF39 and ThRAP2.3* and their proteins.

Gene_ID	Full-length cDNA(bp)	5′UTR (bp)	3′UTR (bp)	ORF (bp)	Predicted peptide				Secondary structure prediction			
					Length	MW (kDa)	PI	GRAVY	Hh (%)	Ee (%)	Tt (%)	Cc (%)
*ThERF15*	1,036	23	194	819	272	31.15	5.02	−0.916	49.63	6.62	0.00	43.75
*ThERF39*	1,723	95	491	1,137	378	41.86	4.93	−0.518	31.48	14.55	0.00	53.97
*ThRAP2.3*	1,410	196	107	1,107	368	41.48	6.20	−0.610	36.41	13.32	0.00	50.27

Corresponding MWs, PIs and grand averages of hydropathicity (GRAVY) for the deduced ThERF15 protein were 31.15 kDa, 5.02 and −0.916, respectively; for ThERF39 were 41.86 kDa, 54.93 and −0.518, respectively; and for ThRAP2.3 were 41.48 kDa, 6.20 and −0.610, respectively (Table 2). Secondary-structure analysis of the three protein sequences, using the GOR IV secondary structure prediction method, revealed similar components in different proportions: alpha helices (Hh)49.63%, 31.48% and 36.41% (in ThERF15, ThERF39 and ThRAP2.3, respectively), extended strands(Ee) 6.62%, 14.55% and 13.32% (in ThERF15, ThERF39 and ThRAP2.3, respectively), and random coils(Cc) 43.75%, 53.97% and 50.27% (in ThERF15, ThERF39 and ThRAP2.3, respectively). All three proteins contained zero% beta turns (Tt) (Table 2). The results of the Prosite analysis suggested that the three proteins had a typical AP2/ERF domain: at amino acid residues 8–141 for ThERF15 (score 22.589), 62–119 for ThERF39 (score 22.748) and 103–160 for ThRAP2.3 (score 58.917).

### Phylogenetic analysis of the ERF proteins

**Figure 2:**
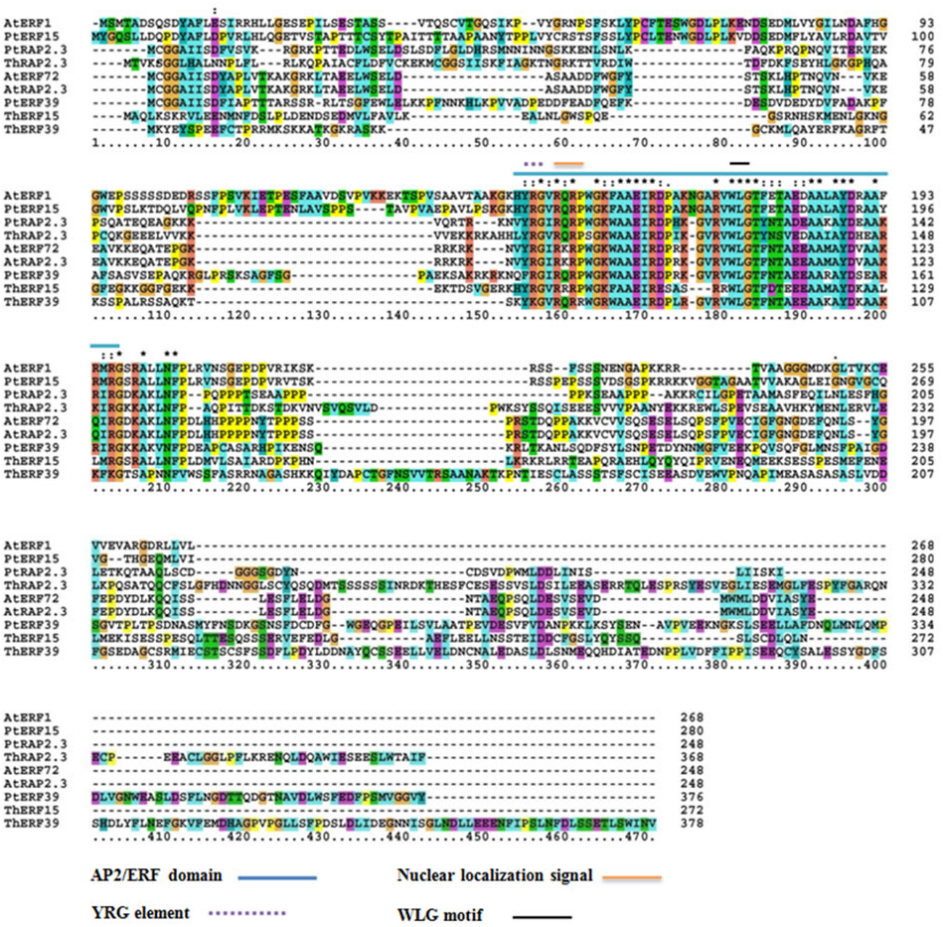
Alignment among the amino acid sequences of ThERF15, ThERF39 and ThRAP2.3 with ERFs of Arabidopsis and Populus protein sequences using the ClustalX program. The AP2-ERF domain, the YRG element, the WLG motif, and the nuclear localization sequence are marked with different kinds of overlines, as indicated above the sequence alignment. Amino acid position numbers are indicated to the right of the sequence.

The protein sequences of ThERF15, ThERF39 and ThRAP2.3 were aligned with *Arabidopsis* protein sequences in The Arabidopsis Information Resource (TAIR; https://www.arabidopsis.org/) (*AtERF1*, AT4G17500.1; *AtERF72*, AT3G16770.1; *AtRAP2.3*, AT3G16770.1) and *Populus* protein sequences in the Joint Genome Institute (JGI: https://jgi.doe.gov/) (*PtERF15*, Potri.003G081200.1; *PtERF39*, Potri.003G071700.1; *PtRAP2.3*, Potri.010G006800.1). The results indicated that ThERF15, ThERF39 and ThRAP2.3 contained a conserved AP2-ERF domain, were members of the plant-specific ERF family of transcription factors and exhibited high levels of sequence homology to one other, including the YRG element, the WLG motif, and the nuclear localization sequence, NLS (Fig. 2).

**Figure 3:**
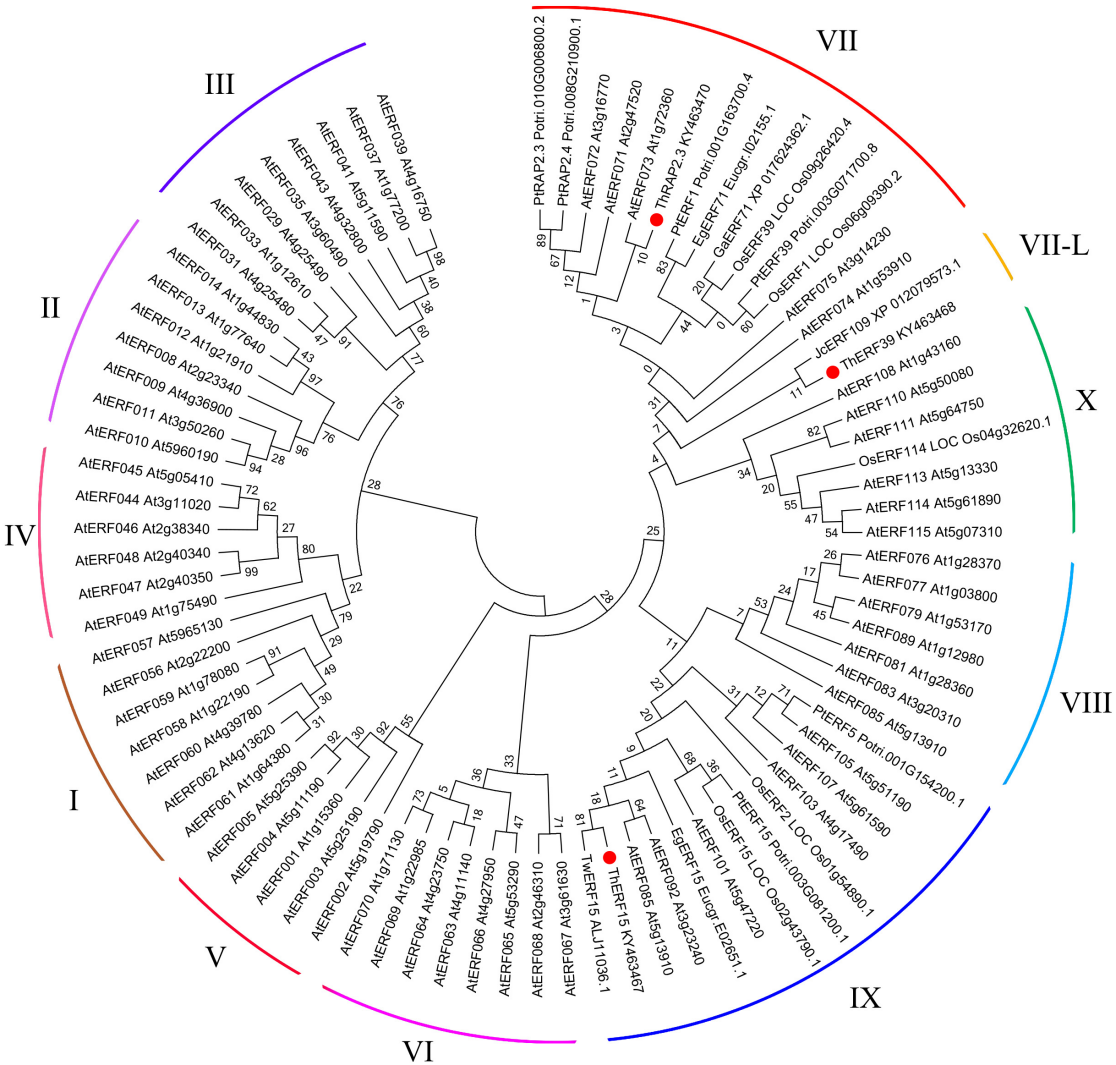
Phylogenetic analysis of the ERF protein family. The phylogenetic tree was constructed using MEGA 7 (http://www.megasoftware.net/), with the maximum likelihood method, using 1,000 replicate bootstrap tests. Numbers near to the nodes indicate bootstrap values obtained from 1,000 replicates. The 84 proteins were clustered into eleven distinct groupsas indicated. At, *Arabidopsis thaliana*; Eg, *Eucalyptus grandis*; Ga, *Gossypium arboretum*; Os, *Oryza sativa*; Pt, *Populus trichocarpa*; Tw, *Taxus wallichiana*; Jc, *Jatropha curcas*.

To explore the evolutionary relationship between ERF proteins from different species, the NJ phylogenetic tree was constructed using ThERF15, ThERF39 and ThRAP2.3 sequences together with the other 81 ERF proteins reported from different plants in the GenBank database, including 65 AtERF proteins (from *Arabidopsis thaliana*) which have been identified and described in detail (Nakano & Suzuki, 2006) (Fig. 3). The un-rooted NJ tree showed that the 84 proteins were clustered into eleven distinct groups (Fig. 3). ThRAP2.3 was positioned in the ERF-VII group, while ThERF15, which was most closely related to TwERF15, was positioned in the ERF-IX group, and ThERF39 was located in the ERF-VII-L (VII-Like) group, due to its relative similarity to the ERF-VII group.

### Expression profiles of the *ERF* genes

To analyze the expression profiles of *ThERF15, ThERF39* and *ThRAP2.3* in ‘Zhongshanshan 406’ under two levels of flooding stress, we carried out qPCR to measure transcript levels at five waterlogging time points, namely 1 d, 3 d, 5 d, 7 d and 9 d, and in different tissues, namely leaves and roots. CK values, being references for two treatments on the same stage and tissue, were set to 1. The results showed that these three *ERF* genes were expressed, at different levels, at each of the five waterlogging time points under HF and TS treatments in roots and leaves of ‘Zhongshanshan 406’.

**Figure 4:**
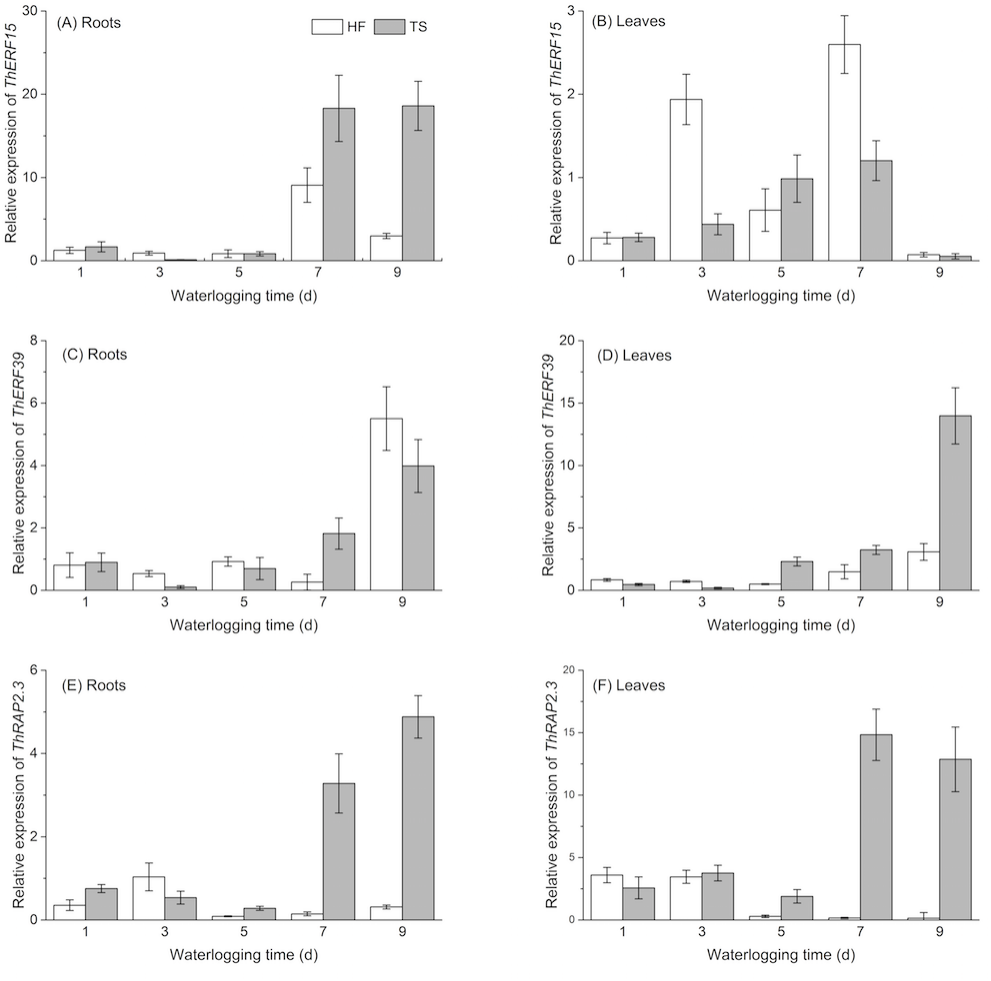
Temporal expression profiles of ThERF15, ThERF39 and ThRAP2.3 in roots and leaves by real-time PCR. HF (half-flooding) and TS (total-submergence) are the two levels of flooding treatment. CK values, being references for two treatments on the same stage and tissue, were set to 1. Data points are the mean values and error bars represent the standard errors, which are from three biological replicates. (A, C, E) are temporal expression profiles of ThERF15, ThERF39 and ThRAP2.3 in roots; (B, D, F) are temporal expression profiles of ThERF15, ThERF39 and ThRAP2.3 in leaves.

Except for *ThERF15* in leaves, the expression levels of all three *ERFs* exhibited a decline (at around day 3 or 5) during the early treatment period, before exhibiting a rise in the late stage of the TS treatment, in both roots and leaves, generally with a maximum expression on the 9th day. Unlike the similar expression patterns in the two tissues under TS treatment, expression patterns in the roots and leaves were significantly different in the HF treatment. *ThERF39* and *ThRAP2.3* both exhibited lower expression levels, compared with CK treatment apart from expression of *ThERF39* on the 9th day, which showed a marked increase. In contrast with the two genes described above, the expression pattern of *ThERF15* had a greater difference in the late period of HF treatment. Significant increases in *ERF* gene expression generally occurred only on days 7 and 9 (Fig. 4).

### Subcellular localization of ERF proteins

**Figure 5:**
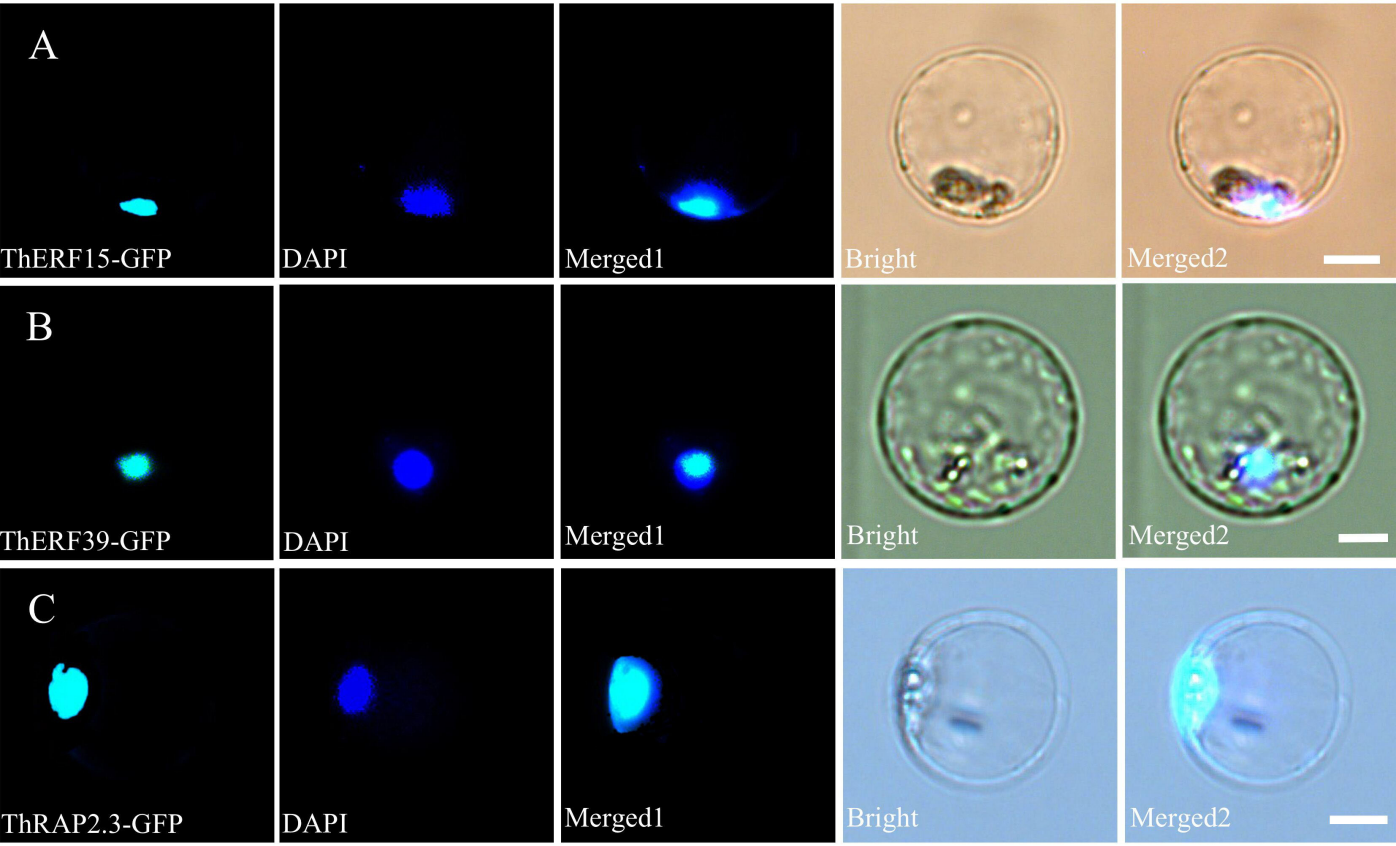
Subcellular localization of ThERF15, ThERF39 and ThRAP2.3 in Populus protoplasts. Transient expression of ThERF15-GFP, ThERF39-GFP and ThRAP2.3-GFP fusion proteins in Populus protoplasts; (A) ThERF15; (B) ThERF39; (C) ThRAP2.3; GFP, fluorescence of ThERF15-GFP, ThERF39-GFP and ThRAP2.3-GFP; DAPI, the protoplasts are stained with DAPI to visualize the nucleus; Merged1, merged images of GFP and DAPI ones; Merged2, merged images of GFP and DAPI ones in bright. Scale bar 5 mm.

To confirm the subcellular localization of ThERF15, ThERF39 and ThRAP2.3 proteins, several GFP-fusion vectors (*35S*::*ThERF15*-*GFP, 35S*:: *ThERF39-GFP and 35S*::*ThRAP2.3-GFP*) were constructed under the control of the double cauliflower mosaic virus 35S (35S CaMV) promoter. Fluorescence signals were observed only in the nucleus when *ThERF15, ThERF39 and ThRAP2.3* were transiently expressed in Populus protoplasts, whilst the nucleuses were identified by DAPI staining data. The result demonstrates that ThERF15, ThERF39 and ThRAP2.3 were each localized to the nucleus (Fig. 5).

## Discussion

In the present study, three *ERFs* from *Taxodium* hybrid ‘Zhongshanshan 406’, with differential expression under waterlogging stress, were isolated and characterized. The deduced proteins of these three *ERFs* contained a typical AP2/ERF domain and hence are considered to belong to the plant-specific ERF subfamily (Li et al., 2017). Multiple sequence alignments revealed that ThERF15, ThERF39 and ThRAP2.3 exhibited considerable protein sequence similarity with other ERFs from *Arabidopsis* and *Populus*, including the AP2-ERF domain, the YRG element, the WLG motif, and the nuclear localization signal (NLS) element. Associated with their role as transcription factors (TFs), all three ThERFs have basic amino acid regions that possibly function as NLSs to target the proteins to the nucleus. The nuclear localization was further verified by transient expression of ThERF15, ThERF39 and ThRAP2.3 in *Populus* protoplasts.

In the meantime, the phylogenetic analysis of plant ERF proteins indicated that ThERF15, ThERF39 and ThRAP2.3 were classified into the ERF-IX, ERF-VII-L and ERF-VII groups, respectively. Studies have shown that ERF proteins act as regulators of plant development and response to biotic and abiotic stresses, including drought, salt and flooding (Quan et al., 2010). Members of the ERF-VII group were confirmed to respond to plant hypoxia and waterlogging stresses and to regulate the expression of related downstream genes in *Arabidopsis* and rice (Hattori et al., 2009; Licausi et al., 2010; Licausi et al., 2010b; Xu et al., 2006), whereas *ThRAP2.3*, which is a member of the ERF-VII group in ‘Zhongshanshan 406’, is extremely likely to be involved in regulation of the waterlogging stress response. However, there are few reports on the flooding-resistant function of members of the ERF-IX and ERF-VII-L groups, and related research needs to be carried out in detail.

Transcript levels of *ThERF15, ThERF39* and *ThRAP2.3* were measured by real-time PCR at five waterlogging time points under the HF and TS treatments. Expression patterns of the three *ERF* genes showed that all three exhibited a sharp increase in expression levels in the latter stages of TS treatment, reaching peak expression on the 9th day, except for *ThERF15* in leaves. Contrary to the results from the TS treatment, the three *ERFs* showed varying expression profiles under the HF treatment. *ThERF39* and *ThRAP2.3* generally had lower expression levels compared with the CK treatment except for *ThERF39* on the 9th day. The expression pattern of *ThERF15* showed greater differences in the later stages of HF treatment. Despite the different expression patterns of the three *ERFs* under the same treatment, all of them exhibited significant increases in expression during the time-course of the two flooding treatments. This indicates that the expression of *ThERF15, ThERF39* and *ThRAP2.3* are clearly influenced by flooding stress, and that the three ERF genes may very possibly be involved in response to flooding stress, especially to extreme flooding stress. The gene-specific response mechanisms and the fine regulation of expression of *ThERF15, ThERF39* and *ThRAP2.3* deserve further study.

In general, the expression of ERF genes is in relation to the molecular response to ethylene. Many ERF TFs are indeed ethylene responsive, despite the observation that the AP2/ERF TFs to which they belong are regulated by numerous physical-chemical stimuli (Licausi et al., 2010; Licausi et al., 2010b). Ethylene has been verified to play a pivotal role in plant responses to biotic and abiotic stresses, including flooding stress (Bleecker & Kende, 2000; Voesenek et al., 2016; Zhang et al., 2016), and these responses are possibly mediated through the ERF gene family, which serve as regulatory elements (Yin et al., 2012). In this study, the concentration of ethylene was determined in the same tissues and treatments as used for the gene expression studies, to explore the regulatory mechanism between plant ethylene accumulation and expression of the three *ERFs* in ‘Zhongshanshan 406’.

Under the HF treatment, the ethylene concentration differed little from that in the CK treatment, although it exhibited an obvious decline during the latter stages of the time-course. During the time-course of the TS treatment, the ethylene concentration in plants under the flooding treatment was always higher than that in plants under the CK treatment. By comparing the expression levels of the three *ERF* s and the trends in ethylene accumulation during flooding stress, it could be seen that ethylene accumulation in roots and leaves showed a clear increase in the early stages of the TS treatment, while the transcription level of *ThRAP2.3* at this point was generally low, increasing, in both leaves and roots, in the later stages of the TS treatment. On the other hand, in comparison with the CK treatment, the accumulation of both ethylene and *ThRAP2.3* mRNA showed a similar trend under the HF treatment, both parameters exhibiting a decline in the later stages of the HF treatment. These findings indicated that*ThRAP2.3* is most likely to be one of the key downstream-response ERF genes to respond to the output of the ethylene signal generated by flooding stress. No direct connection between ethylene content and the transcription level of either *ThERF15* or *ThERF39* could be found. There is a high probability, therefore, that expression of *ThERF15* and *ThERF39* was coordinated by a combination of ethylene and other phytohormones, such as jasmonate (Lorenzo et al., 2003; Sherif et al., 2012). The mechanism of response of expression of *ThERF15* and *ThERF39* to flooding stress, therefore, needs further research.

## Conclusions

Taken together, these results strongly indicate that *ThERF15, ThERF39* and *ThRAP2.3* play an essential part in tolerance to flooding stress in ‘Zhongshanshan 406’, especially *ThRAP2.3*. The combined analysis of ethylene content and expression of the three *ERF* genes indicates that ethylene plays a vital role in response to high flooding stress, with *ThRAP2.3* being one of the key downstream-response ERF genes regulated by the phytohormone ethylene. Further study is necessary to explore the response mechanism of *ThERF15* and *ThERF39* to flooding stress and ethylene accumulation, and to verify the roles in flooding stress tolerance of *ThERF15*, *ThERF39* and *ThRAP2.3*, using genetic transformation, among other strategies.

##  Supplemental Information

10.7717/peerj.4434/supp-1 Supplemental Information 1 Amino acid sequences of three *ERF*s

10.7717/peerj.4434/supp-2 Supplemental Information 2 qPCR

10.7717/peerj.4434/supp-3 Supplemental Information 3 Proteins of ThERFs, AtERFs and other ERFs for Phylogenetic analysis

## Additional Information and Declarations

### DNA Deposition

The following information was supplied regarding the deposition of DNA sequences: Sequences of ThERF15, ThERF39 and ThRAP2.3 described here are accessible via GenBank accession numbers KY463467, KY463468 and KY463470, respectively.

### Data Availability

The following information was supplied regarding data availability: The gene sequences, protein sequences, ethylene concentration and results of qRCR are provided in the Supplemental Files.
