# Supplementary material for: Molecular cloning and expression analysis of three ThERFs involved in the response to waterlogging stress of Taxodium ‘Zhongshanshan406’, and subcellular localization of the gene products
Source: PeerJ. 2018 Mar 12;6:e4434. doi: 10.7717/peerj.4434 (PMC5853676; doi:10.7717/peerj.4434)
Supplement: Supplemental Information 1 [file peerj-06-4434-s001.docx]

>THERF15

GAAAATGGGGATGACATGGTTCAATGGCGCAATTGAAGAGTAAAAGAGTGTTAGAGGAAAATATGAATTTCGATTCCTTACCGCTGGACGAAAACGATTCAGAAGACATGGTTCTGTTTGCAGTTCTTAAGGAAGCCCTGAATCTCGGCTGGTCTCCGCAAGAGGGATCCAGAAATCACTCAAAGATGGAAAATTTGGGGAAAAATGGGGGATTTGAGGGTAAAAAAGGGGGTTTTGGTGAGAAAAAGGAAAAGACGGATTCCGTTGGTGAAAGAAAGCATTACAGAGGCGTGCGCAGACGGCCATGGGGAAAGTTTGCTGCGGAAATCAGAGAATCGGCATCGCGGCGATGGCTGGGGACCTTTGATACGGAAGAGGAAGCCGCCATGGCTTATGATAAAGCTGCCCTGCTCATGAGAGGCTCCCGAGCGCTGTTGAATTTCCCGCTTGATATGGTTCTCAGCGCCATAGCTCGAGACCCTAAGCCTCATAATTTGAAAAGGAAAAGATTGAGGCGTACAGAGGCGCCGCAACGAGCAGAGCATTTGCAGTACCAGTACCAAATACCGAGGGTTGAGAACGAGCAAATGGAGGAAAAATCTGAGTCATCTCCAGAAAGTATGGAATTTGAGAACGAGCTAATGGAGAAAATTTCTGAGTCATCTCCAGAATCTCAGTTAACTACAGAATCTCAATCATCCTCGGAAAGAGTGGAATTTGAGGACCTTGGTGCAGAATTCTTGGAGGAACTGTTGAATTCGTCGACAGAGATTGACGATTGCTTTGGCTCCTTGTATCAGTATAGTTCCCAAAGCCTCTCATGTGACTTACAGCTTAACTAGGCCGCCTTTGTGATTATCTGTTGTATCTTGCCATTTGATGTTTATTGTTACTTTTGCATGGCTTACTCCATTCATTCGGTATAAAGTCTGTACAAAGTGAGTTCATATTAAGAGTCTTTATTGTTCTTTTTAATGTGAATTCATTCACATTCACAAGTATTTATTTAGCTGGGAATTCTTTTGCAAAAAAAAAA

>ThERF39

GAAAATGGGGATTGGTTCAAGAGATCTACATTCCCATAGACTGTCCGCCTGCTGCATCAGATTCTGAAAAGGAATATGAACTTCCCAGTTGCTCCATGAAGTATGAGTACTCACCAGAAGAGTTCTGCACACCTCGCCGTATGAAATCTAAGAAGGCGACCAAAGGGAAGAGGGCTTCTAAGAAGGGATGTAAAATGTTGCAAGCTTATGAACGGTTCAAAGCTGGGAGATTCACCAAATCGAGCCCAGCATTGAGGTCTTCTGCTCAGAAGACATCTAAATATAAGGGAGTTAGACAGAGGAGATGGGGAAGATGGGCTGCTGAGATAAGAGATCCATTAAGAGGAGTTAGAGTATGGTTGGGTACTTTTAATACGGCAGAAGAGGCTGCTAAGGCTTATGACAAGGCAGCTAAGAAATTTAAAGGTACCTCTGCTCCCAATAACTTTGTCTGGTCTTCATTTGCTTCGCGGAGAAATGCTGGAGCTTCACATAAGAAGCAGATCTATGATGCACCCTGCACAGGGTTCAATTCAGTTGTGACAAGGTCTGCTGCAAATGCCAAGACGAAGCCCAATACAATAGAAAGCTGTTTGGCATCATCTTCCACATCTTTTTCCTGTATCTCAGAAGAAGCTTCTGATGTGGAGTGGGTACCAAATCAAGCTCCAATTATGGAAGCCTCTGCATCCGCATCTGCTTCATTGGTTGATGATTTTGGATCTGAAGATGCAGGATGCTCTCGCATGATTGAGTGCAGTACATCTTGTAGCTTCTCTTCAGATTTCTTACCTGATTATTTGGATGACAATGCATATCAATGTTCTAGTGAAGAACTCTTGGTAGAATTAGACAATTGCAATGCCTTAGAGGATGCTTCTCTAGATCTCAGCAACATGGAGCAGCAGCATGACATAGCAACTGAAGATAATCCCCCTCTTGTGGATTTCTTCATTCCTCCTATCAGTGAGGAACAATGTTACAGTGCACTGGAGTCATCATATGGTGATTTTTCAAGTCATGATCTGTATTTCTTGAATGAGTTTGGGAAAGTTTTTGAGATGGATCATGCTGGTCCTGTTCCGGGATTGCTCTCCTTTCCAGATTCTCTTGATCTTATAGATGAAGGGAATAATATCTCTGGTTTAAATGACCTGCTGGAAGAAGAGAATTTCATCCCCAGCTTAAATTTTGATTTGAGTTCTGAAACTCTGAGCTGGATAAATGTATGATTTTTAAAAAACAAGCTCAGGTGGGTTAGGGCGATTTTGTAGATTAGTATCTCAGAGTAATTTCGCAGCAGCCTGTTTTCCCGAGATGATCTTTTGAGCTTATGTGAGGTATATTGACCAAGGTGACAGTCCTTGGCTTACGGTGGATTGTTTATTTTTTAGTATGTAAGACTGAGTTTTGGTTAGGATGGTAGCATATTTCAAGGAACGGAAGAGAGTCATGAGTGAGAATGAATACGGTATTGCCAGTCGGTGACTTGGGTTTTCTTTGTGCTTCAGAGCCCTCCAAGTCATTGACTGTGCTTCCTTCCAAGAAGGAGAAATTGTGCCGTAGCCTTTTATCTCTTGTACCGTGTGGTTTAGTGAATAGGTAATTCTTTATACTTTGTATCCATGGTTGAATCAAAGAAATATGTGTGTGAGCATGAAAAGAGTTTCTTGCTCACTATCAAGTAATACAAGATGGATGATATATTTATGAAAAAAAAAAA

>ThRAP2.3

GAAAATGGGGAATATTGCAAGTTGCAAGACATTTCTTTTAATGTCACATTTATTTTCTTGACAATAAACGTTACATCCAATGCGTAGAGTGGCCCCCACTGCCAATTCAAAGCCCTAATTCTTCCACACAAGTTGCAGATAATCTAGTTTTTAAATCCGCAATAATTAAGGCCACTTGCCTAAAAATTGGGTTTTAATGACGGTAAAAAGCGGAGGTCTGCATGCTTTAAATAATCCCTTGTTTCTAAGGTTGAAACAGCCAGCGATCGCCTGTTTTCTTGATTTTGTCTGCAAAGAGAAAATGTGTGGAGGAAGCATAATATCAAAGTTTATAGCCGGCAAAACTAATGGCCGAAAAACTACTGTCAGAGATATATGGACAGACTTTGACAAATTCTCTGAGTATCATCTTGGTAAAGGTCCTCATCAGGCTCCTTGCCAGAAAGGGGAAGAGGAACTTGTGGTGAAAAAAGTAGTAGAGAAGAAGAGGAAGGCCCATCATTTATATAGAGGAGTGAGGCAGCGTCCTTCTGGAAAATGGGCTGCAGAGATTAGAGACCCCATCAAGGGAGTTAGAGTTTGGCTGGGAACTTACAACTCAGTGGAAGATGCTGCAATTGCCTATGATCATGAGGCTCGAAAGATCAGAGGGAAAAAGGCCAAGCTCAACTTTCCTGCACAACCCATTACCACCGACAAATCTACGGACAAGGTGAATGTCTCTGTTCAATCCGTATTAGATCCATGGAAATCTTATAGCTCTCAGATTTCCGAGGAGGAATCTGTGGTGGTTCCTGCTGCAAATTATGAAAAGAAAAGAGAGTGGCTCAGCCCTGAAGTGTCCGAAGCAGCAGTGCACAAGTATATGGAGAATTTGGAGAGGGTTTTGGAATTGAAACCTCAGAGTGCCACACAACAGTGCTTCTCTCTGGGTTTTCATGACAATAATGGTGGGTTATCTTGTTATCAATCACAAGACATGACGAGCTCGAGCTCGAGCTCGATTAATAGGGACAAAACCCATGAGTCATTTTGTGAATCTGAATCATCTGTATCTTTGGATAGCATTTTGGAAGAGGCATCTGAGCGCAGAACTCAATTGGAATCTCCCAGATCTTATGAAAGCGTGGAGGGTTTGATTGAATCGGAGATGGGTTTGTTTGAATCACCATATTTTGGGGCAAGGCAGAATGAGTGCCCGGAAGAGGCGTGCTTAGGCGGGTTGCCATTCCTGAAGCGAGAGAATCAACTCGACCAGGCATGGATTGAATCCGAGGAAAGTCTGTGGACTGCAATCTTTTGAATCCAGGCTTAATATGGCCTTATCTTTGTTGTATATAGATCTGGATAGAGTGTAGAATAAGTTACTATGTTTTAAATGTTTTGAGTTCTTCTTTCAAAAAAAAAAAA
