# Supplementary material for: Molecular cloning and expression analysis of three ThERFs involved in the response to waterlogging stress of Taxodium ‘Zhongshanshan406’, and subcellular localization of the gene products
Source: PeerJ. 2018 Mar 12;6:e4434. doi: 10.7717/peerj.4434 (PMC5853676; doi:10.7717/peerj.4434)
Supplement: Supplemental Information 3 [file peerj-06-4434-s003.docx]

>56

METASLSFPVPNTSFGVNKSMPLGLNQLTPYQIHQIQNQLNHRRSTISNLSPNRIRMKNLTPSTSKTKNLYRGVRQRHWGKWVAEIRLPKNRTRLWLGTFETAEKAALAYDQAAFQLRGDIAKLNFPNLIHEDMNPLPSSVDTKLQAICKSLRKTEEICSVSDQTKEYSVYSVSDKTELFLPKAELFLPKREHLETNELSNESPRSDETSLLDESQAEYSSSDKTFLDFSDTEFEEIGSFGLRKFPSVEIDWDAISKLANS

>57

MALNMNAYVDEFMEALEPFMKVTSSSSTSNSSNPKPLTPNFIPNNDQVLPVSNQTGPIGLNQLTPTQILQIQTELHLRQNQSRRRAGSHLLTAKPTSMKKIDVATKPVKLYRGVRQRQWGKWVAEIRLPKNRTRLWLGTFETAQEAALAYDQAAHKIRGDNARLNFPDIVRQGHYKQILSPSINAKIESICNSSDLPLPQIEKQNKTEEVLSGFSKPEKEPEFGEIYGCGYSGSSPESDITLLDFSSDCVKEDESFLMGLHKYPSLEIDWDAIEKLF

>58

MTTSMDFYSNKTFQQSDPFGGELMEALLPFIKSPSNDSSAFAFSLPAPISYGSDLHSFSHHLSPKPVSMKQTGTSAAKPTKLYRGVRQRHWGKWVAEIRLPRNRTRLWLGTFDTAEEAALAYDKAAYKLRGDFARLNFPDLRHNDEYQPLQSSVDAKLEAICQNLAETTQKQVRSTKKSSSRKRSSTVAVKLPEEDYSSAGSSPLLTESYGSGGSSSPLSELTFGDTEEEIQPPWNENALEKYPSYEIDWDSILQCSSLVN

>59

MAAAMNLYTCSRSFQDSGGELMDALVPFIKSVSDSPSSSSAASASAFLHPSAFSLPPLPGYYPDSTFLTQPFSYGSDLQQTGSLIGLNNLSSSQIHQIQSQIHHPLPPTHHNNNNSFSNLLSPKPLLMKQSGVAGSCFAYGSGVPSKPTKLYRGVRQRHWGKWVAEIRLPRNRTRLWLGTFDTAEEAALAYDKAAYKLRGDFARLNFPNLRHNGSHIGGDFGEYKPLHSSVDAKLEAICKSMAETQKQDKSTKSSKKREKKVSSPDLSEKVKAEENSVSIGGSPPVTEFEESTAGSSPLSDLTFADPEEPPQWNETFSLEKYPSYEIDWDSILA

>60

MAAIDMFNSNTDPFQEELMKALQPYTTNTDSSSPTYSNTVFGFNQTTSLGLNQLTPYQIHQIQNQLNQRRNIISPNLAPKPVPMKNMTAQKLYRGVRQRHWGKWVAEIRLPKNRTRLWLGTFDTAEEAAMAYDLAAYKLRGEFARLNFPQFRHEDGYYGGGSCFNPLHSSVDAKLQEICQSLRKTEDIDLPCSETELFPPKTEYQESEYGFLRSDENSFSDESHVESSSPESGITTFLDFSDSGFDEIGSFGLEKFPSVEIDWDAISKLSES

>61

MEESNDIFQNNFSPKISEIRASLSQIILAGGPNTLDSIFSLLTPSSVESATTSFNTHNPPPPPQLGSSVYLRQRDIIEKFHLQNRAISTPHPPLFSSTYDHHQTSELMLQAAAGSPAAAFAAALAAGRVTKKKKLYRGVRQRHWGKWVAEIRLPQNRMRVWLGTYDTAEAAAYAYDRAAYKLRGEYARLNFPNLKDPSELLGLGDSSKLIALKNAVDGKIQSICQRVRKERAKKSVKVSKNSSATADSSCLSSPEILSSSPVTTTTTAVTSEDSYWVSPMGLCNSENSSPVSVSVPSEVPATAEEEAMMGVDTDGFLLARMPSFDPELIWEVLAN

>62

MITPIHTQHSLILVYINIYSPPILSKLRTGFILWTNTQKTNKKRNMEDQFPKIETSFMHDKLLSSGIYGFLSSSTPPQLLGVPIFLEGMKSPLLPASSTPSYFVSPHDHELTSSIHPSPVASVPWNFLESFPQSQHPDHHPSKPPNLTLFLKEPKLLELSQSESNMSPYHKYIPNSFYQSDQNRNEWVEINKTLTNYPSKGFGNYWLSTTKTQPMKSKTRKVVQTTTPTKLYRGVRQRHWGKWVAEIRLPRNRTRVWLGTFETAEQAAMAYDTAAYILRGEFAHLNFPDLKHQLKSGSLRCMIASLLESKIQQISSSQVSNSPSPPPPKVGTPEQKNHHMKMESGEDVMMKKQKSHKEVMEGDGVQLSRMPSLDMDLIWDALSFPHSS

>008

METEAAVTATVTAATMGIGTRKRDLKPYKGIRMRKWGKWVAEIREPNKRSRIWLGSYATPEAAARAYDTAVFYLRGPSARLNFPELLAG

LTVSNGGGRGGDLSAAYIRRKAAEVGAQVDALGATVVVNTGGENRGDYEKIENCRKSGNGSLERVDLNKLPDPENSDGDDDECVKRR

>009

METATEVATVVSTPAVTVAAVATRKRDKPYKGIRMRKWGKWVAEIREPNKRSRIWLGSYSTPEAAARAYDTAVFYLRGPSARLNFPELLAGVTVTGGGGGGVNGGGDMSAAYIRRKAAEVGAQVDALEAAGAGGNRHHHHHQHQRGNHDYVDNHSDYRINDDLMECSSKEGFKRCNGSLERVDLNKLPDPETSDDD

>010

MEGGGVADVAVPGTRKRDRPYKGIRMRKWGKWVAEIREPNKRSRLWLGSYSTPEAAARAYDTAVFYLRGPTARLNFPELLPGEKFSDEDMSAATIRKKATEVGAQVDALGTAVQNNRHRVFGQNRDSDVDNKNFHRNYQNGEREEEEEDEDDKRLRSGGRLLDRVDLNKLPDPESSDEEWESKH

>011

MDAGVAVKADVAVKMKRERPFKGIRMRKWGKWVAEIREPNKRSRLWLGSYSTPEAAARAYDTAVFYLRGPTATLNFPELLPCTSAEDMS

AATIRKKATEVGAQVDAIGATVVQNNKRRRVFSQKRDFGGGLLELVDLNKLPDPENLDDDLVGK

>012

MVKQERKIQTSSTKKEMPLSSSPSSSSSSSSSSSSSSCKNKNKKSKIKKYKGVRMRSWGSWVSEIRAPNQKTRIWLGSYSTAEAAARAYDVALLCLKGPQANLNFPTSSSSHHLLDNLLDENTLLSPKSIQRVAAQAANSFNHFAPTSSAVSSPSDHDHHHDDGMQSLMGSFVDNHVSLMDSTSSWYDDHNGMFLFDNGAPFNYSPQLNSTTMLDEYFYEDADIPLWSFN

>013

MVKQELKIQVTTSSSSLSHSSSSSSSSTSALRHQSCKNKIKKYKGVRMRSWGSWVTEIRAPNQKTRIWLGSYSTAEAAARAYDAALLCLKGPKANLNFPNITTTSPFLMNIDEKTLLSPKSIQKVAAQAANSSSDHFTPPSDENDHDHDDGLDHHPSASSSAASSPPDDDHHNDDDGDLVSLMESFVDYNEHVSLMDPSLYEFGHNEIFFTNGDPFDYSPQLHSSEATMDDFYDDVDIPLWSFS

>014

MVKTLQKTPKRMSSPSSSSSSSSSTSSSSIRMKKYKGVRMRSWGSWVSEIRAPNQKTRIWLGSYSTAEAAARAYDAALLCLKGSSANNLNFPEISTSLYHIINNGDNNNDMSPKSIQRVAAAAAAANTDPSSSSVSTSSPLLSSPSEDLYDVVSMSQYDQQVSLSESSSWYNCFDGDDQFMFINGVSAPYLTTSLSDDFFEEGDIRLWNFC

>029

MNSFSAFSEMFGSDYEPQGGDYCPTLATSCPKKPAGRKKFRETRHPIYRGVRQRNSGKWVSEVREPNKKTRIWLGTFQTAEMAARAHDVAALALRGRSACLNFADSAWRLRIPESTCAKDIQKAAAEAALAFQDETCDTTTTNHGLDMEETMVEAIYTPEQSEGAFYMDEETMFGMPTLLDNMAEGMLLPPPSVQWNHNYDGEGDGDVSLWSY

>031

MNSFSAFSEMFGSDYESSVSSGGDYIPTLASSCPKKPAGRKKFRETRHPIYRGVRRRNSGKWVCEVREPNKKTRIWLGTFQTAEMAARAHDVAALALRGRSACLNFADSAWRLRIPESTCAKDIQKAAAEAALAFQDEMCDATTDHGFDMEETLVEAIYTAEQSENAFYMHDEAMFEMPSLLANMAEGMLLPLPSVQWNHNHEVDGDDDDVSLWSY

>033

MNNDDIILAEMRPKKRAGRRVFKETRHPVYRGIRRRNGDKWVCEVREPTHQRRIWLGTYPTADMAARAHDVAVLALRGRSACLNFADSAWRLPVPESNDPDVIRRVAAEAAEMFRPVDLESGITVLPCAGDDVDLGFGSGSGSGSGSEERNSSSYGFGDYEEVSTTMMRLAEGPLMSPPRSYMEDMTPTNVYTEEEMCYEDMSLWSYRY

>035

MGKQINIESSATHHQDNIVSVITATISSSSVVTSSSDSWSTSKRSLVQDNDSGGKRRKSNVSDDNKNPTSYRGVRMRSWGKWVSEIREPRKKSRIWLGTYPTAEMAARAHDVAALAIKGNSGFLNFPELSGLLPRPVSCSPKDIQAAATKAAEATTWHKPVIDKKLADELSHSELLSTAQSSTSSSFVFSSDTSETSSTDKESNEETVFDLPDLFTDGLMNPNDAFCLCNGTFTWQLYGEEDVGFRFEEPFNWQND

>037

MTESSIISVKQSSPVPEEEDHHHHQQDSHRTNTKKRVRSDPGYRGVRMRTWGKWVSEIREPRKKSRIWLGTFSTPEMAARAHDAAALTIKGTSAVLNFPELATYLPRPASSSPRDVQAAAAVAAAMDFSPSSSSLVVSDPTTVIAPAETQLSSSSYSTCTSSSLSPSSEEAASTAEELSEIVELPSLETSYDESLSEFVYVDSAYPPSSPWYINNCYSFYYHSDENGISMAEPFDSSNFGPLFP

>039

MTESSIISVKQSSPVPEEEDHHHHQQDSHRTNTKKRVRSDPGYRGVRMRTWGKWVSEIREPRKKSRIWLGTFSTPEMAARAHDAAALTIKGTSAVLNFPELATYLPRPASSSPRDVQAAAAVAAAMDFSPSSSSLVVSDPTTVIAPAETQLSSSSYSTCTSSSLSPSSEEAASTAEELSEIVELPSLETSYDESLSEFVYVDSAYPPSSPWYINNCYSFYYHSDENGISMAEPFDSSNFGPLFP

>041

MAEEYYSLRSERVTQLLVPNSESDSVSDKSKAEQSEKKTKRGRDSGKHPVYRGVRMRNWGKWVSEIREPRKKSRIWLGTFPTPEMAARAHDVAALSIKGTAAILNFPELADSFPRPVSLSPRDIQTAALKAAHMEPTTSFSSSTSSSSSLSSTSSLESLVLVMDLSRTESEELGEIVELPSLGASYDVDSANLGNEFVFYDSVDYCLYPPPWGQSSEDNYGHGISPNFGHGLSWDL

>043

MADSSSDKEKKENNKQPVYRGVRMRSWGKWVSEIREPRKKSRIWLGTFPTAEMAMRAHDVAAMSIKGTSAILNFPELSKLLPRPVSLSPRDVRAAATKAALMDFDTTAFRSDTETSETTTSNKMSESSESNETVSFSSSSWSSVTSIEESTVSDDLDEIVKLPSLGTSLNESNEFVIFDSLEDLVYMPRWLSGTEEEVFTYNNNDSSLNYSSVFESWKHFP

>044

MAVYEQTGTEQPKKRKSRARAGGLTVADRLKKWKEYNEIVEASAVKEGEKPKRKVPAKGSKKGCMKGKGGPDNSHCSFRGVRQRIWGKWVAEIREPKIGTRLWLGTFPTAEKAASAYDEAATAMYGSLARLNFPQSVGSEFTSTSSQSEVCTVENKAVVCGDVCVKHEDTDCESNPFSQILDVREESCGTRPDSCTVGHQDMNSSLNYDLLLEFEQQYWGQVLQEKEKPKQEEEEIQQQQQEQQQQQLQPDLLTVADYGWPWSNDIVNDQTSWDPNECFDINELLGDLNEPGPHQSQDQNHVNSGSYDLHPLHLEPHDGHEFNGLSSLDI

>045

MAVYDQSGDRNRTQIDTSRKRKSRSRGDGTTVAERLKRWKEYNETVEEVSTKKRKVPAKGSKKGCMKGKGGPENSRCSFRGVRQRIWGKWVAEIREPNRGSRLWLGTFPTAQEAASAYDEAAKAMYGPLARLNFPRSDASEVTSTSSQSEVCTVETPGCVHVKTEDPDCESKPFSGGVEPMYCLENGAEEMKRGVKADKHWLSEFEHNYWSDILKEKEKQKEQGIVETCQQQQQDSLSVADYGWPNDVDQSHLDSSDMFDVDELLRDLNGDDVFAGLNQDRYPGNSVANGSYRPESQQSGFDPLQSLNYGIPPFQLEGKDGNGFFDDLSYLDLEN

>046

MEKEDNGSKQSSSASVVSSRRRRRVVEPVEATLQRWEEEGLARARRVQAKGSKKGCMRGKGGPENPVCRFRGVRQRVWGKWVAEIREPVSHRGANSSRSKRLWLGTFATAAEAALAYDRAASVMYGPYARLNFPEDLGGGRKKDEEAESSGGYWLETNKAGNGVIETEGGKDYVVYNEDAIELGHDKTQNPMTDNEIVNPAVKSEEGYSYDRFKLDNGLLYNEPQSSSYHQGGGFDSYFEYFRF

>047

MPRKRKSRGTRDVAEILRKWREYNEQTEADSCIDGGGSKPIRKAPPKRSRKGCMKGKGGPENGICDYTGVRQRTWGKWVAEIREPGRGAKLWLGTFSSSYEAALAYDEASKAIYGQSARLNLPLLPLCQARLLHFLMNLKFVHVRIQMQDLVLVRSD

>048

MPSEIVDRKRKSRGTRDVAEILRQWREYNEQIEAESCIDGGGPKSIRKPPPKGSRKGCMKGKGGPENGICDYRGVRQRRWGKWVAEIREPDGGARLWLGTFSSSYEAALAYDEAAKAIYGQSARLNLPEITNRSSSTAATATVSGSVTAFSDESEVCAREDTNASSGFGQVKLEDCSDEYVLLDSSQCIKEELKGKEEVREEHNLAVGFGIGQDSKRETLDAWLMGNGNEQEPLEFGVDETFDINELLGILNDNNVSGQETMQYQVDRHPNFSYQTQFPNSNLLGSLNPMEIAQPGVDYGCPYVQPSDMENYGIDLDHRRFNDLDIQDLDFGGDKDVHGST

>049

MSSIEPKVMMVGANKKQRTVQASSRKGCMRGKGGPDNASCTYKGVRQRTWGKWVAEIREPNRGARLWLGTFDTSREAALAYDSAARKLYGPEAHLNLPESLRSYPKTASSPASQTTPSSNTGGKSSSDSESPCSSNEMSSCGRVTEEISWEHINVDLPVMDDSSIWEEATMSLGFPWVHEGDNDISRFDTCISGGYSNWDSFHSPL

>001

MVQTKKFRGVRQRHWGSWVAEIRHPLLKRRIWLGTFETAEEAARAYDEAAVLMSGRNAKTNFPLNNNNTGETSEGKTDISASSTMSSSTSSSSLSSILSAKLRKCCKSPSPSLTCLRLDTASSHIGVWQKRAGSKSDSSWVMTVELGPASSSQETTSKASQDAILAPTTEVEIGGSREEVLDEEEKVALQMIEELLNTN

>002

MEHQTTPKQKTKEKSKGNKTKFVGVRQRPSGKWVAEIKDTTQKIRMWLGTFETAEEAARAYDEAACLLRGSNTRTNFANHFPNNSQLSLKIRNLLHQKQSMKQQQQQQHKPVSSLTDCNINYISTATSLTTTTTTTTTTAIPLNNVYRPDSSVIGQPETEGLQLPYSWPLVSGFNHQIPLAQAGGETHGHLNDHYSTDQHLGLAEIERQISASLYAMNGANSYYDNMNAEYAIFDPTDPIWDLPSLSQLFCPT

>003

MARPQQRFRGVRQRHWGSWVSEIRHPLLKTRIWLGTFETAEDAARAYDEAARLMCGPRARTNFPYNPNAIPTSSSKLLSATLTAKLHKCYMASLQMTKQTQTQTQTQTARSQSADSDGVTANESHLNRGVTETTEIKWEDGNANMQQNFRPLEEDHIEQMIEELLHYGSIELCSVLPTQTL

>004

MVHSRKFRGVRQRQWGSWVSEIRHPLLKRRVWLGTFETAEAAARAYDQAALLMNGQNAKTNFPVVKSEEGSDHVKDVNSPLMSPKSLSELLNAKLRKSCKDLTPSLTCLRLDTDSSHIGVWQKRAGSKTSPTWVMRLELGNVVNESAVDLGLTTMNKQNVEKEEEEEEAIISDEDQLAMEMIEELLNWS

>005

MVHSKKFRGVRQRQWGSWVSEIRHPLLKRRVWLGTFDTAETAARAYDQAAVLMNGQSAKTNFPVIKSNGSNSLEINSALRSPKSLSELLNAKLRKNCKDQTPYLTCLRLDNDSSHIGVWQKRAGSKTSPNWVKLVELGDKVNARPGGDIETNKMKVRNEDVQEDDQMAMQMIEELLNWTCPGSGSIAQV

>063

METEKKVSLPRILRISVTDPYATDSSSDEEEEVDFDALSTKRRRVKKYVKEVVLDSVVSDKEKPMKKKRKKRVVTVPVVVTTATRKFRGVRQRPWGKWAAEIRDPSRRVRVWLGTFDTAEEAAIVYDNAAIQLRGPNAELNFPPPPVTENVEEASTEVKGVSDFIIGGGECLRSPVSVLESPFSGESTAVKEEFVGVSTAEIVVKKEPSFNGSDFSAPLFSDDDVFGFSTSMSESFGGDLFGDNLFADMSFGSGFGFGSGSGFSSWHVEDHFQDIGDLFGSDPVLTV

>064

MEAEKKMVLPRIKFTEHKTNTTTIVSELTNTHQTRILRISVTDPDATDSSSDDEEEEHQRFVSKRRRVKKFVNEVYLDSGAVVTGSCGQMESKKRQKRAVKSESTVSPVVSATTTTTGEKKFRGVRQRPWGKWAAEIRDPLKRVRLWLGTYNTAEEAAMVYDNAAIQLRGPDALTNFSVTPTTATEKKAPPPSPVKKKKKKNNKSKKSVTASSSISRSSSNDCLCSPVSVLRSPFAVDEFSGISSSPVAAVVVKEEPSMTTVSETFSDFSAPLFSDDDVFDFRSSVVPDYLGGDLFGEDLFTADMCTDMNFGFDFGSGLSSWHMEDHFQDIGDLFGSDPLLAV

>065

MDEYIDFRPLKYTEHKTSMTKYTKKSSEKLSGGKSLKKVSICYTDPDATDSSSDEDEEDFLFPRRRVKRFVNEITVEPSCNNVVTGVSMKDRKRLSSSSDETQSPASSRQRPNNKVSVSGQIKKFRGVRQRPWGKWAAEIRDPEQRRRIWLGTFETAEEAAVVYDNAAIRLRGPDALTNFSIPPQEEEEEEEPEPVIEEKPVIMTTPTPTTSSSESTEEDLQHLSSPTSVLNHRSEEIQQVQQPFKSAKPEPGVSNAPWWHTGFNTGLGESDDSFPLDTPFLDNYFNESPPEMSIFDQPMDQIFCENDDIFNDMLFLGGETMNIEDELTSSSIKDMGSTFSDFDDSLISDLLVA

>066

MMMDEFMDLRPVKYTEHKTVIRKYTKKSSMERKTSVRDSARLVRVSMTDRDATDSSSDEEEFLFPRRRVKRLINEIRVEPSSSSTGDVSASPTKDRKRINVDSTVQKPSVSGQNQKKYRGVRQRPWGKWAAEIRDPEQRRRIWLGTFATAEEAAIVYDNAAIKLRGPDALTNFTVQPEPEPVQEQEQEPESNMSVSISESMDDSQHLSSPTSVLNYQTYVSEEPIDSLIKPVKQEFLEPEQEPISWHLGEGNTNTNDDSFPLDITFLDNYFNESLPDISIFDQPMSPIQPTENDFFNDLMLFDSNAEEYYSSEIKEIGSSFNDLDDSLISDLLLV

>067

MERRTRRVKFTENRTVTNVAATPSNGSPRLVRITVTDPFATDSSSDDDDNNNVTVVPRVKRYVKEIRFCQGESSSSTAARKGKHKEEESVVVEDDVSTSVKPKKYRGVRQRPWGKFAAEIRDPSSRTRIWLGTFVTAEEAAIAYDRAAIHLKGPKALTNFLTPPTPTPVIDLQTVSACDYGRDSRQSLHSPTSVLRFNVNEETEHEIEAIELSPERKSTVIKEEEESSAGLVFPDPYLLPDLSLAGECFWDTEIAPDLLFLDEETKIQSTLLPNTEVSKQGENETEDFEFGLIDDFESSPWDVDHFFDHHHHSFD

>068

MKSRVRKSKYTVHRKITSTPFDGFPKIVKIIVTDPCATDSSSDEENDNKSVAPRVKRYVDEIRFCDEDDEPKPARKAKKKSPAAAAENGGDLVKSVVKYRGVRQRPWGKFAAEIRDPSSRTRLWLGTFATAEEAAIGYDRAAIRIKGHNAQTNFLTPPPSPTTEVLPETPVIDLETVSGCDSARESQISLCSPTSVLRFSHNDETEYRTEPTEEQNPFFLPDLFRSGDYFWDSEITPDPLFLDEFHQSLLPNINNNNTVCDKDTNLSDSFPLGVIGDFSSWDVDEFFQDHLLDK

>069

MKRIVRISFTDMEATDSSSSEDESPPSSRRRGKKLVKEIVIDHSDPPEVGKTRFKIRIPASLLAARNTTANKKKFRGVRQRPWGKWAAEIRCGRVKGRPERIWLGTFETAEEAALAYDNAAIQLIGPDAPTNFGRPDVDSAVVKKQDSDASGGASEEVV

>070

MKRIIRISFTDAEATDSSSDEDTEERGGASQTRRRGKRLVKEIVIDPSDSADKLDVCKTRFKIRIPAEFLKTAKTEKKYRGVRQRPWGK

WVAEIRCGRGACKGRRDRLWLGTFNTAEEAALAYDNASIKLIGPHAPTNFGLPAENQEDKTVIGASEVARGA

>071

MCGGAIISDFIWSKSESEPSQLGSVSSRKKRKPVSVSEERDGKRERKNLYRGIRQRPWGKWAAEIRDPSKGVRVWLGTFKTADEAARAYDVAAIKIRGRKAKLNFPNTQVEEEADTKPGGNQNELISENQVESLSEDLMALEDYMRFYQIPVADDQSATDIGNLWSYQDSN

>072

MCGGAIISDYAPLVTKAKGRKLTAEELWSELDASAADDFWGFYSTSKLHPTNQVNVKEEAVKKEQATEPGKRRKRKNVYRGIRKRPWGKWAAEIRDPRKGVRVWLGTFNTAEEAAMAYDVAAKQIRGDKAKLNFPDLHHPPPPNYTPPPSSPRSTDQPPAKKVCVVSQSESELSQPSFPVECIGFGNGDEFQNLSYGFEPDYDLKQQISSLESFLELDGNTAEQPSQLDESVSEVDMWMLDDVIASYE

>073

MCGGAVISDYIAPEKIARSSGKSSWRSNGVFDCSIYDFDGNFDELESDEPFVFSSTHKHHASGSASDGKKKQSSRYKGIRRRPWGRWAAEIRDPIKGVRVWLGTFNTAEEAARAYDLEAKRIRGAKAKLNFPNESSGKRKAKAKTVQQVEENHEADLDVAVVSSAPSSSCLDFLWEENNPDTLLIDTQWLEDIIMGDANKKHEPNDSEEANNVDASLLSEELLAFENQTEYFSQMPFTEGNCDSSTSLSSLFDGGNDMGLWS

>074

MCGGAIISDFIPPPRSRRVTSEFIWPDLKKNLKGSKKSSKNRSNFFDFDAEFEADFQGFKDDSSIDCDDDFDVGDVFADVKPFVFTSTPKPAVSAAAEGSVFGKKVTGLDGDAEKSANRKRKNQYRGIRQRPWGKWAAEIRDPREGARIWLGTFKTAEEAARAYDAAARRIRGSKAKVNFPEENMKANSQKRSVKANLQKPVAKPNPNPSPALVQNSNISFENMCFMEEKHQVSNNNNNQFGMTNSVDAGCNGYQYFSSDQGSNSFDCSEFGWSDQAPITPDISSAVINNNNSALFFEEANPAKKLKSMDFETPYNNTEWDASLDFLNEDAVTTQDNGANPMDLWSIDEIHSMIGGVF

>075

MCGGAIISDFIPPPRSLRVTNEFIWPDLKNKVKASKKRSNKRSDFFDLDDDFEADFQGFKDDSAFDCEDDDDVFVNVKPFVFTATTKPVASAFVSTGIYLVGSAYAKKTVESAEQAEKSSKRKRKNQYRGIRQRPWGKWAAEIRDPRKGSREWLGTFDTAEEAARAYDAAARRIRGTKAKVNFPEEKNPSVVSQKRPSAKTNNLQKSVAKPNKSVTLVQQPTHLSQQYCNNSFDNSFGDMSFMEEKPQMYNNQFGLTNSFDAGGNNGYQYFSSDQGSNSFDCSEFGWSDHGPKTPEISSMLVNNNEASFVEETNAAKKLKPNSDESDDLMAYLDNALWDTPLEVEAMLGADAGAVTQEEENPVELWSLDEINFMLEGDF

>076

MAPTVKTAAVKTNEGNGVRYRGVRKRPWGRYAAEIRDPFKKSRVWLGTFDTPEEAARAYDKRAIEFRGAKAKTNFPCYNINAHCLSLTQSLSQSSTVESSFPNLNLGSDSVSSRFPFPKIQVKAGMMVFDERSESDSSSVVMDVVRYEGRRVVLDLDLNFPPPPEN

>077

MTTEKENVTTAVAVKDGGEKSKEVSDKGVKKRKNVTKALAVNDGGEKSKEVRYRGVRRRPWGRYAAEIRDPVKKKRVWLGSFNTGEEAARAYDSAAIRFRGSKATTNFPLIGYYGISSATPVNNNLSETVSDGNANLPLVGDDGNALASPVNNTLSETARDGTLPSDCHDMLSPGVAEAVAGFFLDLPEVIALKEELDRVCPDQFESIDMGLTIGPQTAVEEPETSSAVDCKLRMEPDLDLNASP

>079

MPNITMGLKPDPVAPTNPTHHESNAAKEIRYRGVRKRPWGRYAAEIRDPVKKTRVWLGTFDTAQQAARAYDAAARDFRGVKAKTNFGVIVGSSPTQSSTVVDSPTAARFITPPHLELSLGGGGACRRKIPLVHPVYYYNMATYPKMTTCGVQSESETSSVVDFEGGAGKISPPLDLDLNLAPPAE

>081

MASTTCAREVHYRGVRKRPWGRYAAEIRDPWKKTRVWLGTFDTPEEAALAYDGAARFLRGIKAKTNFPSPLSLDLNHLPSAPSAATAAANNQPHQHQQLWFAAPPPVPPSSDHHHQHHRIFLRTGVLNDKTSDYSSTEAPLYFTSSPNTATSSPGYQVVGFPMMNSSPSPVTVRRGLAIDLNEPPPLWL

>083

MRKGRGSSVVGPALPVTAGGSVKEPRYRGVRKRPWGRFAAEIRDPLKKSRVWLGTFDSAVDAARAYDTAARNLRGPKAKTNFPIDCSPSSPLQPLTYLHNQNLCSPPVIQNQIDPFMDHRLYGGGNFQEQQQQQIISRPASSSMSSTVKSCSGPRPMEAAAASSSVAKPLHAIKRYPRTPPVAPEDCHSDCDSSSSVIDDGDDIASSSSRRKTPFQFDLNFPPLDGVDLFAGGIDDLHCTDLRL

>089

MEKALRNFTESTHSPDPNPLTKFFTEPTASPVSRNRKLSSKDTTVTIAGAGSSTTRYRGVRRRPWGRYAAEIRDPMSKERRWLGTFDTAEQAACAYDSAARAFRGAKARTNFTYPTAVIMPEPRFSFSNKKSSPSARCPLPSLPLDSSTQNFYGAPAAQRIYNTQSIFLRDASCSSRKTTPYNNSFNGSSSSYSASKTACVSYSENENNESFFPEESSDTGLLQEVVQEFLKKNRGVPPSPPTPPPVTSHHDNSGYFSNLTIYSENMVQETKETLSSKLDRYGNFQANDDGVRAVADGGLSLGSNEWGYQEMLMYGTQLGCTCRRSWG

>091

MAFGNIQELDGEILKNVWANYIGTPQTDTRSIQVPEVSRTWEALPTLDDIPEGSREMLQSLDMSTEDQEWTEILDAIASFPNKTNHDPLTNPTIDSCSLSSRVSCKTRKYRGVRKRPWGKFAAEIRDSTRNGVRVWLGTFQTAEEAAMAYDKAAVRIRGTQKAHTNFQLETVIKAMEMDCNPNYYRMNNSNTSDPLRSSRKIGLRTGKEAVKAYDEVVDGMVENHCALSYCSTKEHSETRGLRGSEETWFDLRKRRRSNEDSMCQEVEMQKTVTGEETVCDVFGLFEFEDLGSDYLETLLSSF

>092

MDPFLIQSPFSGFSPEYSIGSSPDSFSSSSSNNYSLPFNENDSEEMFLYGLIEQSTQQTYIDSDSQDLPIKSVSSRKSEKSYRGVRRRPWGKFAAEIRDSTRNGIRVWLGTFESAEEAALAYDQAAFSMRGSSAILNFSAERVQESLSEIKYTYEDGCSPVVALKRKHSMRRRMTNKKTKDSDFDHRSVKLDNVVVFEDLGEQYLEELLGSSENSGTW

>093

MEYSQSSMYSSPSSWSSSQESLLWNESCFLDQSSEPQAFFCPNYDYSDDFFSFESPEMMIKEEIQNGDVSNSEEEEKVGIDEERSYRGVRKRPWGKFAAEIRDSTRNGIRVWLGTFDKAEEAALAYDQAAFATKGSLATLNFPVEVVRESLKKMENVNLHDGGSPVMALKRKHSLRNRPRGKKRSSSSSSSSSNSSSCSSSSSTSSTSRSSSKQSVVKQESGTLVVFEDLGAEYLEQLLMSSC

>101

MYGQCNIESDYALLESITRHLLGGGGENELRLNESTPSSCFTESWGGLPLKENDSEDMLVYGLLKDAFHFDTSSSDLSCLFDFPAVKVEPTENFTAMEEKPKKAIPVTETAVKAKHYRGVRQRPWGKFAAEIRDPAKNGARVWLGTFETAEDAALAYDIAAFRMRGSRALLNFPLRVNSGEPDPVRITSKRSSSSSSSSSSSTSSSENGKLKRRRKAENLTSEVVQVKCEVGDETRVDELLVS

>103

MATPNEVSALFLIKKYLLDELSPLPTTATTNRWMNDFTSFDQTGFEFSEFETKPEIIDLVTPKPEIFDFDVKSEIPSESNDSFTFQSNPPRVTVQSNRKPPLKIAPPNRTKWIQFATGNPKPELPVPVVAAEEKRHYRGVRMRPWGKFAAEIRDPTRRGTRVWLGTFETAIEAARAYDKEAFRLRGSKAILNFPLEVDKWNPRAEDGRGLYNKRKRDGEEEEVTVVEKVLKTEESYDVSGGENVESGLTAIDDWDLTEFLSMPLLSPLSPHPPFGYPQLTVV

>105

MASSHQQQQEQDQSALDLITQHLLTDFPSLDTFASTIHHCTTSTLSQRKPPLATIAVPTTAPVVQENDQRHYRGVRRRPWGKYAAEIRDPNKKGVRVWLGTFDTAMEAARGYDKAAFKLRGSKAILNFPLEAGKHEDLGDNKKTISLKAKRKRQVTEDESQLISRKAVKREEAQVQADACPLTPSSWKGFWDGADSKDMGIFSVPLLSPCPSLGHSQLVVT

>107

METFEESSDLDVIQKHLFEDLMIPDGFIEDFVFDDTAFVSGLWSLEPFNPVPKLEPSSPVLDPDSYVQEILQMEAESSSSSSTTTSPEVETVSNRKKTKRFEETRHYRGVRRRPWGKFAAEIRDPAKKGSRIWLGTFESDIDAARAYDYAAFKLRGRKAVLNFPLDAGKYDAPVNSCRKRRRTDVPQPQGTTTSTSSSSSN

>108

MVSMLTNVVSGETEPSASATWTMGHKREREEFSLPPQPLITGSAVTKECESSMSLERPKKYRGVRQRPWGKWAAEIRDPHKATRVWLGTFETAEAAARAYDAAALRFRGSKAKLNFPENVGTQTIQRNSHFLQNSMQPSLTYIDQCPTLLSYSRCMEQQQPLVGMLQPTEEENHFFEKPWTEYDQYNYSSFG

>110

MSAMVSALTQVVSARSQTEAEGAHSSSSSAGHKRGWLGIDSAPIPSSFARVDSSHNPIEESMSKAFPEEAREKKRRYRGVRQRPWGKWAAEIRDPHRAARVWLGTFDTAEAAARAYDEAALRFRGNKAKLNFPEDVRILPPPPPLLRSPADTVANKAEEDLINYWSYTKLLQSSGQRSFLERGQEESSNIFEHSPMEQPLPPSSSGPSSSNFPAPSLPNT

>111

MCVLKVANQEDNVGKKAESIRDDDHRTLSEIDQWLYLFAAEDDHHRHSFPTQQPPPSSSSSSLISGFSREMEMSAIVSALTHVVAGNVPQHQQGGGEGSGEGTSNSSSSSGQKRRREVEEGGAKAVKAANTLTVDQYFSGGSSTSKVREASSNMSGPGPTYEYTTTATASSETSSFSGDQPRRRYRGVRQRPWGKWAAEIRDPFKAARVWLGTFDNAESAARAYDEAALRFRGNKAKLNFPENVKLVRPASTEAQPVHQTAAQRPTQSRNSGSTTTLLPIRPASNQSVHSQPLMQSYNLSYSEMARQQQQFQQHHQQSLDLYDQMSFPLRFGHTGGSMMQSTSSSSSHSRPLFSPAAVQPPPESASETGYLQDIQWPSDKTSNNYNNSPSS

>113

MVSALSRVIENPTDPPVKQELDKSDQHQPDQDQPRRRHYRGVRQRPWGKWAAEIRDPKKAARVWLGTFETAEEAALAYDRAALKFKGTKAKLNFPERVQGPTTTTTISHAPRGVSESMNSPPPRPGPPSTTTTSWPMTYNQDILQYAQLLTSNNEVDLSYYTSTLFSQPFSTPSSSSSSSQQTQQQQLQQQQQQREEEEKNYGYNYYNYPRE

>114

MYGKRPFGGDESEEREEDENLFPVFSARSQHDMRVMVSALTQVIGNQQSKSHDNISSIDDNYPSVYNPQDPNQQVAPTHQDQGDLRRRHYRGVRQRPWGKWAAEIRDPKKAARVWLGTFETAESAALAYDEAALKFKGSKAKLNFPERVQLGSNSTYYSSNQIPQMEPQSIPNYNQYYHDASSGDMLSFNLGGGYGSGTGYSMSHDNSTTTAATTSSSSGGSSRQQEEQDYARFWRFGDSSSSPHSGY

>115

MANSGNYGKRPFRGDESDEKKEADDDENIFPFFSARSQYDMRAMVSALTQVIGNQSSSHDNNQHQPVVYNQQDPNPPAPPTQDQGLLRKRHYRGVRQRPWGKWAAEIRDPQKAARVWLGTFETAEAAALAYDNAALKFKGSKAKLNFPERAQLASNTSTTTGPPNYYSSNNQIYYSNPQTNPQTIPYFNQYYYNQYLHQGGNSNDALSYSLAGGETGGSMYNHQTLSTTNSSSSGGSSRQQDDEQDYARYLRFGDSSPPNSGF

>ThERF15

MAQLKSKRVLEENMNFDSLPLDENDSEDMVLFAVLKEALNLGWSPQEGSRNHSKMENLGKNGGFEGKKGGFGEKKEKTDSVGERKHYRGVRRRPWGKFAAEIRESASRRWLGTFDTEEEAAMAYDKAALLMRGSRALLNFPLDMVLSAIARDPKPHNLKRKRLRRTEAPQRAEHLQYQYQIPRVENEQMEEKSESSPESMEFENELMEKISESSPESQLTTESQSSSERVEFEDLGAEFLEELLNSSTEIDDCFGSLYQYSSQSLSCDLQLN

>ThERF39

MKYEYSPEEFCTPRRMKSKKATKGKRASKKGCKMLQAYERFKAGRFTKSSPALRSSAQKTSKYKGVRQRRWGRWAAEIRDPLRGVRVWLGTFNTAEEAAKAYDKAAKKFKGTSAPNNFVWSSFASRRNAGASHKKQIYDAPCTGFNSVVTRSAANAKTKPNTIESCLASSSTSFSCISEEASDVEWVPNQAPIMEASASASASLVDDFGSEDAGCSRMIECSTSCSFSSDFLPDYLDDNAYQCSSEELLVELDNCNALEDASLDLSNMEQQHDIATEDNPPLVDFFIPPISEEQCYSALESSYGDFSSHDLYFLNEFGKVFEMDHAGPVPGLLSFPDSLDLIDEGNNISGLNDLLEEENFIPSLNFDLSSETLSWINV

>ThRAP2.3

MTVKSGGLHALNNPLFLRLKQPAIACFLDFVCKEKMCGGSIISKFIAGKTNGRKTTVRDIWTDFDKFSEYHLGKGPHQAPCQKGEEELVVKKVVEKKRKAHHLYRGVRQRPSGKWAAEIRDPIKGVRVWLGTYNSVEDAAIAYDHEARKIRGKKAKLNFPAQPITTDKSTDKVNVSVQSVLDPWKSYSSQISEEESVVVPAANYEKKREWLSPEVSEAAVHKYMENLERVLELKPQSATQQCFSLGFHDNNGGLSCYQSQDMTSSSSSSINRDKTHESFCESESSVSLDSILEEASERRTQLESPRSYESVEGLIESEMGLFESPYFGARQNECPEEACLGGLPFLKRENQLDQAWIESEESLWTAIF

>OsERF15

MLLNPASREVAALDSIRHHLLEEEEETPATAPAPTRRPVYCRSSSFGSLVADQWSESLPFRPNDAEDMVVYGALRDAFSSGWLPDGSFAAVKPESQDSYDGSSIGSFLASSSSEAGTPGEVTSTEATVTPGIREGEGEAVAVASRGKHYRGVRQRPWGKFAAEIRDPAKNGARVWLGTFDSAEEAAVAYDRAAYRMRGSRALLNFPLRIGSEIAAAAAAAAAGNKRPYPDPASSGSSSPSSSSSSSSSSSSGSPKRRKRGEAAAASMAMALVPPPPPPAQAPVQLALPAQPWFAAGPIQQLVS

>OsERF114

MVTALAHVIRAAPDLHLPHHPSSSASAAAHPQQASSFYPTAAAAASSPSDQLAAAAAAEEQGRRRHYRGVRQRPWGKWAAEIRDPKKAARVWLGTFDTAEDAAIAYDEAALRFKGTKAKLNFPERVQGRTDLGFLVTRGIPPAATHGGGYYPSSSPAAGACPPPRQQQTVVPYPDLMRYAQLLQGGVGGSYMPFGGAATMSSSTVSSSSAPQILDFSTQQLIRAGPPSPMPSSGSGSATAAASSTTSASSPGAWPYGGSERKKKDSSS

>OsERF2

MSLSLGFSAGAGVGADRLAAAPALQAAGALPPRVDVSLSLARAANGQPSSYLPLNENDSLDMVLFDVLREASAVAALSSSSSSSPELGARTTAPVVAGHPAGRKGGGGGGGGRGAAARGGAAGGRHYRGVRRRPWGKYAAEIRDPTRHGARLWLGTFGTAEEAAAAYDRAAFRMRGAKALLNFPPAVAGDGARRGAAAAAKQVGMSDVVPRACHVVCYSVLSAHIFYDIFRGVIPIP

>EgERF15

MVPPFPTAELPLNENDSQDMVIYHVLNEAMSQNNSSLPHPNQSGSPSSGGSLEPSRGITKKHYRGVRRRPWGKFAAEIRDSLRHGA

RVWLGTFETAEEAALAYDRAAFRMRGAKALLNFPPEVVAASMPMERYEPSRSQSPGSANSDTRGQTHRSDSEVGIAGGASRDVATDHLEKVLDVF

>PtERF15

MYGQSLLDQPDYAFLDPVRLHLQGETVSTAPTTTCSYTPAITTTTAAPAANYTPPLVYCRSTSFSSLYPCLTENWGDLPLKVDDSEDMFLYAVLRDAVTVGWVPSLKTDQLVQPNFPLVKLEPTENLAVSPPSTAVPVAEPAVLPSKGKHYRGVRQRPWGKFAAEIRDPAKNGARVWLGTFE

TAEDAALAYDRAAYRMRGSRALLNFPLRVNSGEPDPVRVTSKRSSPEPSSSVDSGSPKRRKKVGGTAGAATVVAKAGLEIGNGVGCQVGTHGEQMLVI

>PtERF5 Potri.001G154200.1

MSTDVSTALEFIKHHLLGDLLSPIATSSSASFCQFSTNTEISTYETTGCCSQASTSDSSTAFPYFLDSPGPDFFVFSSDFSPAQDNKTNIFEFEAKPEIIDLLTPKPLDSTSHLNSQPSSSSSSSNSSNLFEFVKPQIISHPVNNFFYEESKPRVEPTRKPSLKISLPSRKSEWIQFSNTNP

QPVDDNSGVAVEEKKHYRGVRHRPWGKYAAEIRDPNRRGSRVWLGTFDTALEAARAYDRAAFKLRGSKAILNFPLEAGRCDVRANEEGERKRLRECDAEEREDVKRVMRVVKREEPERDVPLTPSCCTAAWDICGDSKGVFNVPPLSPLSPHPSLGFPQLLVI

>OsERF39 LOC_Os09g26420.4

MLTTKLVQHDGPTARSAKHKRKNQYRGIRQRPWGKWAAEIRDPSKGVRVWLGTYNTAEEAARAYDAEARKIRGKKAKVNFPDEPAVAQKLSLKQNAAKQEKLAPPLKTCGDDAFFQLNSSDNDLFAMLAKVPAKPAEPVDLMPPVKPLASTETFEMNMLSDTSSNSFGSSDFGWEDDTLTPDYTSVFVPNAAMPAYGEPAYLTGGAPKRMRNNYGIAVPQGNGMPNLAQNMPTFDPEMKYLPLPYVESSSDESMDNLLQNDATQDGASNEGIWSLDELLMAAGAY

>OsERF1 LOC_Os06g09390.2

MCGGAILSDLIPPPRRVTAGDLWLEKTKKQQQQKKKNKGARRLPLRQEEEDDFEADFEEFEVDSGEWEVESDADEAKPLAAPRSGFAKGGLKNTTVAGADGPAARSAKRKRKNQFRGIRQRPWGKWAAEIRDPRKGVRVWLGTFNSPEEAARAYDAEARRIRGKKAKVNFPDGAPVASQRSHAEPSSMNMPAFSIEEKPAVMSAGNKTMYNTNAYAYPAVEYTLQEPFVQIQNVSFVPAMNAIEDTFVNLSSDQGSNSFGCSDFSQENDIKTPDITSMLAPTMTGVDDSAFLQNNASDAMVPPVMGNASIDLADLEPYMKFLIDGGSDESIDTLLSSDGSQDVASSMDLWSFDDMPVSAEFY

>EgERF71

QCVGAPSSPTSSRRLTSDFLWPDLKRSAGKQSRRPARSEVVDVVDDDFEADFQGFKDESDVEDDFDDEVEVDVKPFAFSAAEPRYSKGSSTTKSVEYNGQAEKSAKRKRKNQYRGIRQRPWGKWAAEIRDPRKGVRVWLGTFNTAEEAARAYDAEARRIRGKKAKVNFPDDSSSASSKRSVKSNVQKLPKTTTNNVQPNLNQNFNYANSSDDDIYSSMGFVEEKPPTNQFYMDALNAQGVSGMNSLSPADSAPLYFNSDQGSNSFECSDFGWGENGPRTPDVSSVLSATLEVDESQFEDANPRKKIRSASDDVSEEENTAAKTFSEELSAFESDMKFFQMPFVDGGWDPSVEALLGGEATQDGGNAVDLWSFDDLAPMMGGVF

>PtERF39

MCGGAIISDFIAPTTTARSSRRLTSGFEWLELKKPFNNKHLKPVVADPEDDFEADFQEFKDESDVDEDYDVFADAKPFAFSASVSEPAQKRGLPRSKSAGFSGPAEKSAKRKRKNQFRGIRQRPWGKWAAEIRDPRKGVRVWLGTFNTAEEAARAYDSEARRIRGKKAKVNFPDEAPCASAR

HPIKENSQKRLTKANLSQDFSYLSNPETDYNNMGFVEEKPQVSQFGLMNSFPAIGDSGVTPLTPSDNASMYFNSDKGSNSFDCDFGWGEQGPEILSVLAATPEVDESVFVDANPKKLKSYSENALP

>PtERF1

MCGGAIISDFIPPTTTARSSRRFAEGFEWFGMKKPLDNKKYSKPVVINLDDDFEADFQEFKDESDVDENYDVFVDAKPFSFSATASAPAKKRSAPVKSAEFSGQAEKSAKRKRKNQYRGIRQRPWGKWAAEIRDPRKGVRVWLGTFNTAEEAARAYDAEARRIRGKKAKVNFPDEAPLASSK

QSIKENSRKPLPKTNSSQSFSYLSNPELNYNNMGFVEEKPLVNQFGSMNSFPVNGDSGMKTLAPSDSAPMCFNSDQGSNSFDCDFGWGEQTPMTPESLTVLAATPEADEYLLANPEELESYSENAVPVEEKNGKSLSEELLAFDSQLKYLQMPDAEGSSWEASLDSFLNVETTQDGTNAMDLWSFDDFPSMVGGVY

>PtRAP2.3

MCGGAIISDFVSVKRGRKPTTEDLWSELDSLSDFLGLDHRSMNNINNGSKKENLSNLKFAQKPRQPNQERVEKPSQATEQEAGKKKVQRTRKNVYRGIRQRPWGKWAAEIRDPHKGVRVWLGTYNTADEAAKAYDEAAKRIRGDKAKLNFPPQPPPTSEAAPPPPPKSEAAPPPAKKRCILG

PETAAMASFEQILNLESFHGLETKQTAAQLSCDGGGSGDYNCDSVDPWMLDDLINISLIISKI

>PtRAP2.4

MCGGAIISDFVTVKRGRRLTVEDLWSELDPFSEFLRFDYHSNDNNGSKKDPSNLLFPQKPSYTIQVQQVITEKVEKPSHATEKENGNKKAQRTRKSVYRGIRQRTWGKWAAEIRDPHKGARVWLGTYNTAEEAAKAYDEAAKRIRGDKAKLNFPSQTPPTPEAPPPAKKRCIVAPETAYVAS

PFTPPLQEPYSGYQNEDYELEEQILKLESFLGLEPDQMAARLRENGGDNRDSGDLWMLDDLVTHHQYRRQINY

>TwERF15 ALJ11036.1

MEQSKENLGKIASSSEVSFPLNENDSEDMVLYGVLKEAVTLGWSGFPEHEDPRSKPEKLTDGEQKKSKEAIAGPKKRYRGVRRRPWGKYAAEIRDSTSRRWLGTFDTEEEAAMAYDRAAFTMRGSRANLNFPLDKVVAAIAPPNFENDSVPGDLTRKRLRRHTDHTQKQEQEQEMDKTSQSTQVMELEDLGADYLDELLRSSSKIHDCHGFWDMECPH

>GaERF71 XP_017624362.1

MCGGAIISEFIPRKGGRRVTAADIWPNSPFVKINGFCSNRDHSVCHFKRSQPEPPSSLAVDEQVEKKPKRQRKNLYRGIRQRPWGKWAAEIRDPRKGVRVWLGTFNTAEEAARAYDREARKIRGKKAKVNFPNEDDSFSTPYNLNRSTHNSSPCVYQQHINSNYGFNYDLNEIGGYATDPIVISGDENKGSDDQNVNLCYVPVKAEELEEEEKRREPMNKAVSAVGVQEENNEVQKLSEELMAYENYMRFYQIPYLDGESPTQNGVAPQDSVVGELWSFDDDGVAVPETSTAM

>JcERF109 XP_012079573.1

MNITTNINRLTSEEEDSIMVAALKQVISGDTSSIAPPNIIHILQGSDKCPVCNMDSVDCLGCNFFGSNQEEKDDSNKERMNKKKNKYRGVRQRPWGKWAAEIRDPWRAIRVWLGTFSTAEEAARAYDTAAIKFRGNKAKTNFPLSDYAQTSKNNEKMEKYKNAGESSSKVVAEEDDSIDSMMIED
